# Combined Effect of the Potassium Dose and Plant Biofertilization by *Acinetobacter calcoaceticus* on the Growth, Mineral Content, Nutritional Quality, Antioxidant Activity, and Metabolomic Features of Tomatillo Fruits (*Physalis ixocarpa* Brot.)

**DOI:** 10.3390/plants12030466

**Published:** 2023-01-19

**Authors:** Heriberto F. Ramírez-Cariño, Carlos E. Ochoa-Velasco, José A. Guerrero-Analco, Juan L. Monribot-Villanueva, Concepción Calderón-García, Elizabeth González-Terreros, Cirenio Escamirosa-Tinoco, Isidro Morales, Rogelio Valadez-Blanco

**Affiliations:** 1 Instituto Politécnico Nacional, Centro Interdisciplinario de Investigación para el Desarrollo Integral Regional—Unidad Oaxaca, Hornos 1003, Santa Cruz Xoxocotlán, Oaxaca 71230, Mexico; 2 Departamento de Bioquímica-Alimentos, Facultad de Ciencias Químicas, Benemérita Universidad Autónoma de Puebla, Puebla 72420, Mexico; 3 Laboratorio de Química de Productos Naturales, Red de Estudios Moleculares Avanzados, Instituto de Ecología A. C., Clúster Científico y Tecnológico BioMimic®, Carretera Antigua a Coatepec 351, El Haya, Xalapa, Veracruz 91073, Mexico; 4 Instituto de Agroindustrias, Universidad Tecnológica de la Mixteca, Carretera a Acatlima km. 2.5, Huajuapan de León, Oaxaca 69000, Mexico; 5 Instituto de Estudios Ambientales, Universidad de la Sierra Juárez, Avenida Universidad S/N, Ixtlán de Juárez, Oaxaca 68725, Mexico

**Keywords:** rhizobacterial biofertilizer, plant-growth promoting rhizobacteria, nutritional quality of agro-products, husk tomato crop cultivation, potassium fertilization, sustainable agriculture, plant-rhizobacteria metabolomics

## Abstract

An *Acinetobacter calcoaceticus* UTMR2 strain was evaluated in tomatillo plants (*Physalis ixocarpa* Brot.) using a factorial design with different potassium doses (100, 75, 50 and 0% of the recommended dose). In addition to the agronomic parameters, an analysis of the physicochemical, antioxidant, and metabolomic properties of the fruit was performed. The application of the inoculant affected several parameters of the plant (chlorophyll, weight, and contents of several mineral elements) as well as of the fruit (yield, maturity index, FRAP antioxidant capacity, and contents of protein, fiber, and fat). A multivariate analysis was performed by means of a PCA and a heatmap, indicating that the inoculant induced a strong modulating activity in tomatillo plants for the evaluated parameters, with a remarkable effect at low K doses (0 and 50%). The inoculated treatment at 75% of the K dose resulted in similar plant and fruit characteristics to the fully fertilized control. On the other hand, the biofertilized treatment with no K addition resulted in the highest values in the plant and fruit parameters. In addition, from the metabolomics analysis of the fruits at 75% of the K dose, the up-regulation of 4,4″-bis(N-feruloyl)serotonin, salvianolic acid K, and chlorogenic acid was observed, which may have a role in anti-senescence and resistance mechanisms. In conclusion, the rhizobacterial strain had a positive effect on plant growth, nutritional quality, bioactive compounds, and antioxidant activity of tomatillo fruits at reduced doses of K fertilizer, which gives support for its consideration as an effective biofertilizer strain.

## 1. Introduction

Tomatillo, husk tomato, or green tomato (*Physalis ixocarpa* Brot.) is one of the most cultivated vegetables in the American Continent due to its highly appreciated sensorial properties and its high nutritional value, containing 8.24 °Brix, 0.75–1.06% protein, 1.12–2.10% fat and 0.77–1.42% ash [1]. Tomatillo fruits are mainly rich in potassium, and they are also a rich source of Mg, Ca, Na, P, and bioactive compounds [2]. In 2021, the cultivated area of tomatillo crops in Mexico was about 42,673 ha, with a production of about 824,977 tons [3].

To increase the tomatillo plant’s growth and productivity, it is necessary to improve the soil quality by the addition of essential nutrients to the plants [4]. For this reason, it is known that a large number of chemical fertilizers have been used extensively despite the fact that the extensive use of these chemicals has negative effects on the environment and on the soil’s sustainability. The use of rhizobacterial biofertilizers, also known as plant-growth-promoting rhizobacteria (PGPR), constitutes an alternative for reducing the use of chemical fertilizers aiming at developing sustainable crop cultivation systems.

The beneficial effects of rhizobacteria on crop production have been attributed mainly to a number of mechanisms for nutrient assimilation and chemical signaling. Direct biofertilization mechanisms improve nutrient availability and uptake by the plant, which have a direct effect on the plant’s growth. The main direct mechanisms include the following: N_2_ fixation; P, K, and Zn solubilization; and the production of siderophores and phytohormones. On the other hand, indirect mechanisms (ISR and ACC deaminase) improve the tolerance of the plants against stress factors, which can be both biotic (pathogens) and abiotic (drought, salinity, and heavy metal toxicity) [5].

Improvements in plant growth, crop yield, and quality of a wide range of fruit crops with the use of rhizobacterial inoculants have been widely documented in the literature [6]. A few studies in the literature have reported the effect of rhizobacterial application in tomatillo crop cultivation (*Physalis ixocarpa* Brot.) on the growth parameters of seedlings and plants. Rojas-Solis et al. [7] reported that the application of *Pseudomonas fluorescens* strains alone and in combination with *Bacillus thuringiensis* resulted in significant beneficial effects on the development of tomatillo seedlings, in comparison with the non-inoculated control (fresh weight, hypocotyl, and root length). In a similar study, Hernández-Pacheco et al. [8] reported that the rhizobacterial consortium comprised of *Microbacterium oxydans*, *Stenotrophomonas maltophilia*, *B. toyonensis*, *Microbacterium foliorum*, *Leifsonia shinshuensis*, and *Neobacillus drentensis* increased the length of the primary and lateral roots, the fresh weight of root and stem, and the total weight of tomatillo plants (*P. ixocarpa*) in comparison with the non-inoculated treatment. Similarly, in our previous work on tomatillo biofertilization [9], we reported that the putative biofertilizer strains *Atlantibacter* sp., *Priestia megaterium,* and *Acinetobacter calcoaceticus* increased the dry leaf weight (>349%), root length (>11%), dry root weight (>479%), and plant height (>140%) of tomatillo seedlings in comparison with the non-inoculated control. In addition, it was reported that the strain *A. calcoaceticus* increased the concentration of three minerals: K (37%), Ca (80%), and Mg (81%), in comparison with the non-inoculated seedlings. Considering these results, in the present work, it was decided to continue the study of the biofertilization effect of the *A. calcoaceticus* UTMR2 strain on tomatillo fruit.

*A. calcoaceticus* is a species found in natural places, such as soil, fresh water, sediments, and contaminated areas. This species has been reported as a phosphorus, potassium, and zinc solubilizer as well as a nitrogen fixer [10]. The beneficial growth-promoting traits attributed to the rhizobacterial strain *A. calcoaceticus* have been reported in the literature. Yamakawa et al. [11] reported that this species increased the growth of duckweed (*Lema minor*) from 1.5- to 2-fold compared with the non-inoculated control. In addition, the co-inoculation of this species with *Pseudomonas* sp. increased the growth of the plant 2.3-fold. Similarly, Sadiq and Ali [12] observed that *A. calcoaceticus* significantly increased the shoot and root length and the roots/plant in wheat (*Triticum sativum*) in comparison with the non-inoculated treatment, whereas no effect was observed on the growth and yield of *T. aestivum* at full maturity. In another study, Foughalia et al. [13] reported that the root and shoot length (99 and 43%, respectively), the root and shoot fresh weight (69 and 102%, respectively), and the number of leaves of tomato seedlings were significantly enhanced by the application of *A. calcoaceticus* compared with the untreated controls. In a similar study, Li et al. [14] reported that the application of a rhizobacterial consortium comprised of *Providencia rettgeri*, *Advenella incenata*, *A. calcoaceticus*, and *Serratia plymuthica* significantly increased the dry weight, plant height, root length, and root surface area of oat, alfalfa, and cucumber seedlings.

To date, there are no reports on the effect of rhizobacteria on the yield and nutritional quality of tomatillo fruits. In contrast, due to the worldwide utilization of tomato crops, several biofertilization studies on this vegetable have been reported in the literature. In a study by Yagmur and Gunes [15], the inoculants *B. megaterium, Paenibacillus polymxa*, *Azospirillum* sp., and *Burkholderia cepacia* increased tomato crop yield. In another study, Lee et al. [16] reported that the application of *Rhodopseudomonas palustris* increased the vitamin C (3×), lycopene (19%), and total phenolic compounds (16%) compared with the non-inoculated control. In a similar study, Katsenios et al. [17] evaluated the biofertilization effect of nine rhizobacterial strains on the cultivation of industrial tomatoes. The application of *B. subtilis*, *B. amyloliquefaciens*, *Priestia megaterium*, and *B. licheniformis* increased the mean fruit weight per plant, with the latter also causing an increase in yield per plant. In terms of the nutritional quality of the fruits, *B. pumilus* increased the total soluble solids, while *P. megaterium* improved the contents of lycopene and total carotenoids, with most of the bacterial strains causing an increase in the antioxidant activity of the fruit. Ochoa-Velasco et al. [18] evaluated the effect of the rhizobacterial inoculant *B. licheniformis* on the nutritional quality of tomato fruits at different doses of N fertilization. The authors reported that the bacterial inoculant had a positive effect on the synthesis of flavonoids by the plant at a 75% of the recommended nitrogen dose. The biofertilizer mainly contributed to the vitamin C and lycopene contents in the fruit. On the other hand, very few metabolomic studies have been reported regarding the biofertilization effects on fruits. A biofertilization study on tomatoes was performed by Fatima et al. [19] in which the effect of the inoculation of *Pseudomonas aeruginosa* on the nutritional quality of tomato fruits was assessed.

Considering the above, the objective of this work was to evaluate the effect of the application of the rhizobacteria *Acinetobacter calcoaceticus* UTMR2 at different doses of potassium on the plant agronomic parameters, as well as on the fruit quality traits, such as mineral content, bioactive compounds, antioxidant activity, and the metabolic profile of the most relevant biofertilization treatments on tomatillo plants (*Physalis ixocarpa* Brot.).

## 2. Results

### 2.1. Mineral Content in Plants

For the mineral content in tomatillo plants (Table 1), there were no clear significant differences in K concentration between the treatments, with the inoculated treatment at 0% of the K dose (KB0) resulting in a higher K content than its counterpart without inoculation (KF0). In addition, the inoculant caused an increase in the P content at low K doses (0 and 50%) in tomatillo plants in comparison with the corresponding treatments without inoculation, whereas the inoculated treatments at 100 and 75% K doses resulted in the lowest P concentrations. On the other hand, the treatments with no K fertilization with and without inoculation resulted in the highest Ca, Mg, and Mn content in the plants, whereas the treatments with the full K dose presented the lowest content of these elements. The highest Na concentration in the plants was observed in the inoculated treatment with the full K dose (KB100), whereas the biofertilized treatment without K fertilization had the lowest concentration of this element.

### 2.2. Plant Physiological Parameters

The photosynthetic activity and chlorophyll content during crop cultivation was monitored (Table 2). No significant differences were found between the treatments for the photosynthetic activity. On the other hand, in the chlorophyll determinations, the lower doses of K (0 and 50), in combination with the inoculation of the bacteria, presented the highest chlorophyll values (176.9 and 164.6 mmol/m^2^, respectively), which were statistically similar to the biofertilized treatment with a 75% K dose.

### 2.3. Plant Growth

The dry weights of the plants for the different treatments varied from 42.6 to 79.8 g (Table 2). The treatment with K fertilization at 50% with inoculant (KB50) resulted in the highest dry weight (50% higher than its non-biofertilized counterpart), whereas the non-inoculated treatment with 75% of the K dose (KF75) resulted in the lowest dry weight. On the other hand, the inoculated treatment at a K dose of 50% (KB50) resulted in a higher plant height with respect to the rest of the treatments, which were statistically similar among them. In addition, no differences in plant stem thickness were observed among the treatments (Table 2).

### 2.4. Production Yield

The fruit diameter (polar and equatorial) was not affected by the applied treatments (Table 3). The fruit yield per tomatillo plant was determined at the end of the harvest period. The application of *A. calcoaceticus* inoculant increased the fruit yield (287.2 g/plant) in the KB0 treatment in comparison with its non-inoculated counterpart (215.3 g/plant). The inoculated treatment with a K dose of 75% (KB75) resulted in the highest fruit yield parameters (g/plant and g/fruit) among the treatments, which were even larger than that of the non-inoculated plants with the full K dose (KF100). In addition, these treatments (KB75 and KF100) presented the largest number of fruits per plant. In addition, the biofertilized treatment with no K addition (KB0) resulted in overall yield values that were among the highest between the treatments. The highest overall yield index was observed in the inoculated treatment with a 75% K dose. In the case of the treatment with no K fertilization, the inoculated plants had a higher yield index than the non-biofertilized control, with the opposite effect for the treatments that were fully K-fertilized.

### 2.5. Quality Characteristics of the Harvested Tomatillo Fruits

Color measurements were made 15 days after the harvest of the tomatillo fruits. No clear trend could be found in the color parameters L*, a*, and b* for the different treatments (Table S1). In the proximal analysis of tomatillo fruits (Table 4), the total soluble solids (TSS) content ranged from 5.5 to 6.1 °Brix for the inoculated treatments without K fertilization (KB0) and the 75% K dose (KF75), respectively. Not a clear effect on the TSS of the non-inoculated treatments was observed. However, for the biofertilized plants, TSS increased with the K dose. The titrable acidity (TA) for tomatillo treatments varied from 0.59 to 0.94. The highest pH was determined in the treatment with a 50% dose of K without inoculant (KF50) (pH = 4.1) with respect to the rest of the treatments. The non-inoculated treatment without K addition had the lowest pH value (3.8). In terms of the sugar:acid ratio (TSS:TA), which will be termed maturity index (MI) in this work, there was not a clear pattern among the treatments. The inoculated treatment at a 100% dose of K (KB100) resulted in the highest MI value (10.0), which was considerably higher than the non-fertilized counterpart (KF100, 7.3), whereas the lowest values for this parameter were obtained in the treatments with 75% of the K dose (KB75) and that with no K fertilization without inoculant (KF0).

In terms of the protein content in fruits, the values ranged from 0.6 to 2.0%, with the treatments KF75, KB100, and KB75 resulting in the highest protein contents. The biofertilized plants with a 50% dose (KB50) and null K fertilization (KB0) presented the lowest protein contents among the treatments. The fat content in tomatillo fruits ranged from 0.38 to 0.86%. For this component, not a clear trend was observed in the non-inoculated treatments. However, in the biofertilized plants, lower fat contents were obtained at low K doses (0 and 50%) in comparison with those at high K doses (75 and 100%). The fiber content in the fruits varied from 1.0 to 2.1%. The inoculated treatments at low K doses (KB0 and KB50) presented the highest fiber contents, which values were significantly higher than those observed in the treatments without rhizobacterial inoculation at 50 and 75% K doses. The lowest fiber content was observed in the non-biofertilized treatment with a 50% K dose. The ash content ranged from 0.51 to 0.64%. There was not a clear trend in the ash content of the different treatments. Similarly, no significant differences were observed in the electric conductivity among the treatments.

### 2.6. Mineral Content in Tomatillo Fruit

The contents of K, P, Mg, Mn, and the electric conductivity (EC) in tomatillo fruits were not affected either by the fertilizer dose or by the application of the inoculant. On the other hand, no clear trend was observed for Ca and Na contents in the plants for the different treatments (Table S2). Regarding the Na content, the fully fertilized treatments, with and without inoculant, resulted in the lowest Na content, whereas the non-inoculated treatment had a higher Na concentration than the biofertilized plants. The non-fertilized treatment had a higher content of Ca in comparison with the inoculated plants.

### 2.7. Phenols Content and Antioxidant Capacity in Tomatillo Fruits

**Figure 1:**
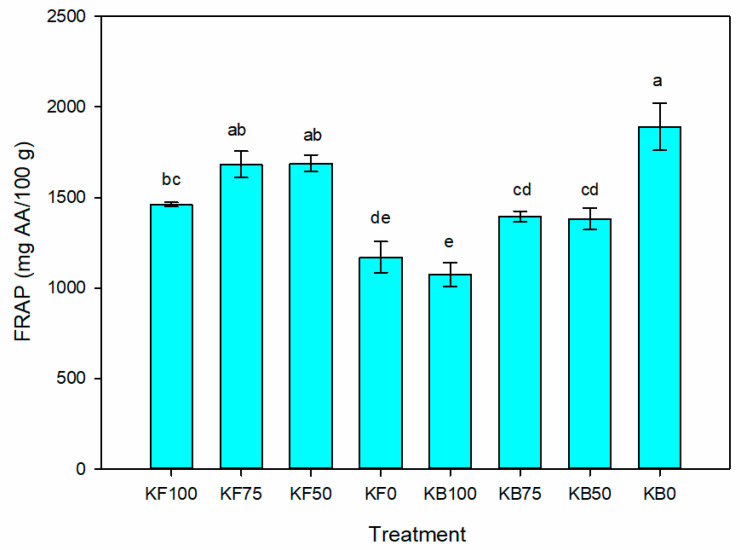
Antioxidant capacity (FRAP) of tomatillo fruits (*Physalis ixocarpa* Brot. cv. “cáscara morada”) in the evaluation of the biofertilizing effect of *Acinetobacter calcoaceticus* UTMR2 at different doses of potassium. Concentration in dry-weight basis (mg ascorbic acid (AA)/100 g dw). KF#: treatments with chemical fertilization only; KB#: treatments with chemical fertilization and biofertilization; #: potassium fertilization dose (%) based on the recommended dose for tomatillo cultivation [20]. Different letters in the columns indicate a significant difference between the treatments according to the HSD Tukey test (*p* < 0.05, n = 3).

The total phenolic compounds (TPC) and antioxidant capacity of the DPPH and FRAP assays were determined (Figure S1). The TPC content and the antioxidant capacity of the fruit samples by the DPPH and the FRAP assays were in the range from 162.7 to 270.4 mg of gallic acid equivalents (GAE) 100 g dw, from 212.7 to 249.8 mg Trolox/100 g dw, and from 1075.2 to 1891.1 mg of ascorbic acid (AA)/100 g dw, respectively. No significant differences were obtained for the TPC and DPPH values. On the other hand, in the biofertilized treatments, the ferric-reducing antioxidant power (FRAP) decreased with the K dose in the plants with no K addition resulting in the highest antioxidant capacity among the treatments (Figure 1).

### 2.8. Multivariate Analysis

**Figure 2:**
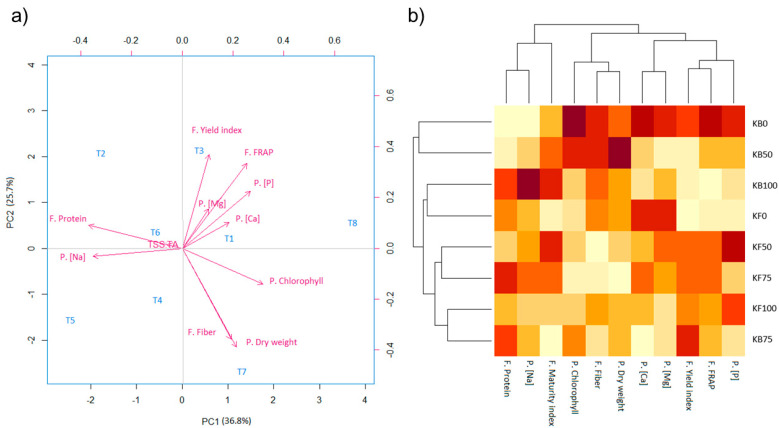
Multivariate analysis for the relevant parameters of tomatillo plants and fruits (*Physalis ixocarpa* Brot. cv. “cáscara morada”) in the evaluation of the biofertilizing effect of *Acinetobacter calcoaceticus* UTMR2 at different doses of potassium. P: plant parameter; F: fruit parameter. T#: treatments according to the general experimental design; KF#: treatments with chemical fertilization only; KB#: treatments with chemical fertilization and biofertilization; #: potassium fertilization dose (%) based on the recommended dose for tomatillo cultivation [20]. (**a**) Principal component analysis (PCA biplot); (**b**) heatmap visualization plot.

For the multivariate analysis, we selected the parameters that showed a significant difference for the treatments considered in this work in which the fertilization dose of potassium and rhizobacterial inoculation were assessed. Six parameters were considered for the plant analysis: chlorophyll, dry weight, and contents of Na, Ca, P, and Mg; and five for the fruit: protein, fiber, as well as maturity and yield indexes, and FRAP antioxidant capacity. From the PCA study (Figure 2a), a total coverage of 62.5% of the variance was obtained (Figure S2), in which the PC1 had a 36.8% coverage, while the PC2 had 25.7% of the remaining variance of the properties tested. Additionally, in the heatmap (Figure 2b) it can be seen that the treatment that obtained the highest values in most of the parameters measured was T8, followed by T7, which corresponded to the biofertilized treatments with low K doses (0 and 50%, respectively). Following these, treatment T5 presented the highest values of Na plant content and maturity index, and treatment T4 presented the high contents of Ca and Mg in the plants. Treatments T1, T2, T3, and T6 had similar characteristics between them and can be subgrouped into pairs since similar results were obtained in yield index, FRAP, and fiber for treatments T2 and T3, while for treatments T1 and T6, similar results were obtained in the plant’s Mg content and dry weight of the plant.

### 2.9. Untargeted and Targeted Metabolomics

**Figure 3:**
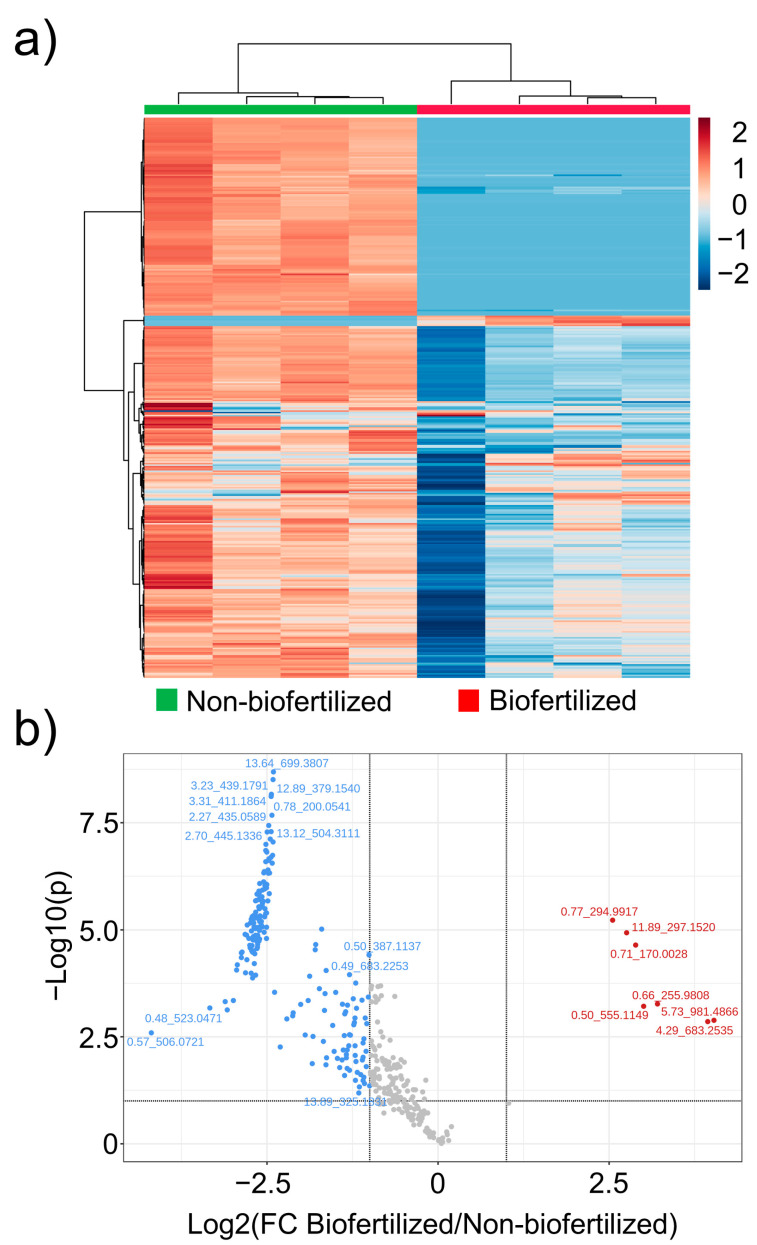
(**a**) Heatmap of biofertilized (KB75) and non-biofertilized (KF75) samples analyzed in ESI-mode in the evaluation of the biofertilizing effect of *Acinetobacter calcoaceticus* UTMR2 in tomatillo fruits (*Physalis ixocarpa* Brot cv. “cáscara morada”) at 75% of the recommended potassium dose [20]. At the top is shown the hierarchical clustering using the algorithm of Ward and the Euclidean distance. (**b**) Volcano plot representing the results of the fold-change analysis. Both figures were obtained using the MetaboAnalyst bioinformatic platform (https://www.metaboanalyst.ca/MetaboAnalyst/home.xhtml, accessed on 7 September 2022).

In order to study the effect of biofertilization with the rhizobacterial strain *A. calcoaceticus* in tomatillo plants, we performed untargeted and targeted metabolomics based on mass spectrometry. For this, we selected the treatment with 75% of the recommended dose of K with and without biofertilization. The comparative untargeted metabolomic analysis performed in tomatillo fruits allowed us to determine important chemical differences between both treatments. In Figure 3a, a heatmap is used to depict the clustering of the chemical profiles of the samples for both treatments. From the heatmap, it can be observed that most of the signals (mass/charge ratios; m/z) were over-accumulated in the non-inoculated treatment, whereas over-accumulation was only observed with a few signals in the biofertilized treatment (Figure 3a). This tendency was confirmed with a fold-change analysis represented by a volcano plot (Figure 3b), in which it was found that only seven m/z signals exhibited fold-change values higher than two in the biofertilizated treatment compared with the non-inoculated control. In contrast, the non-biofertilized treatment exhibited 201 m/z signals with fold-change values higher than two compared with the biofertilizated treatment. The compound 4,4′’-bis(N-feruloyl)serotonin was tentatively identified from the m/z signals with a biofertilized/non-biofertilized change value of 15.4, as well as salvianolic acid K, with a fold-change value of eight (Table 5). In contrast, several compounds from different chemical groups were tentatively identified as over-accumulated in the non-biofertilized treatment: organic acids, such as acetamidobutanoic acid, citric acid, hydroxybutyric acid, N-acetylglutamic acid, and citraconic acid; phenolics, such as trihydroxy-methyl-diprenylxanthone, quercetin-rhamnoside-glucoside, ferulic acid, and sinapic acid; the amino acids L-tryptophan, L-aspartic acid, and L-glutamic acid; the fatty acid hydroperoxylinoleic acid; and the chlorophyll precursor magnesium protoporphyrin (Table 5).

In addition, a metabolomic analysis targeting phenolics compounds was performed. Eight phenolic compounds belonging to the phenolic acid and flavonoid groups were identified and quantified in the samples, plus the amino acid precursor phenylalanine (Table 6). In addition, rhizobacterial inoculation increased the concentration of chlorogenic acid and decreased the concentration of the phenolic acids *t*-cinnamic acid, ferulic acid, protocatechuic acid, 4-coumaric acid, sinapic acid, and the flavonoids quercetin-3-glucoside and quercetin 3,4′-di-O-glucoside (Table 6). The amino acid precursor phenylalanine, the phenolic acid vanillic acid, and the flavonoid rutin exhibited no statistical differences among the treatments (Table 6).

## 3. Discussion

### 3.1. Effect on Mineral Content in Plant

From the analysis of the water and soil used in this work, the soil had an organic matter content of 2.0% [21], which may act as a carbon source for the rhizobacterial inoculant, thus contributing to its colonization and growth. In addition, the soil had a moderately alkaline pH (8.0), an electrical conductivity of 1.4 dS/m, a very high supply of available P (202 ppm), a considerable supply of inorganic N (58.1 ppm), a medium supply of available K (261 ppm), a medium available Mg (332 ppm), a low available Fe and Mn (4.67 and 3.32 ppm, respectively), and a cation exchange capacity of 23.1 me/100 g. On the other hand, the pH of the water was of a medium salinity (7.4), a high electrical conductivity (1.7 dS/m), a medium K content, a moderately high Ca and Mg content, and a hardness of 41.6 °f, which is considered very hard [21].

From the ANOVA results (Table S3), both factors (K dose and inoculation), individual and combined, had a significant effect on the dry weight and the contents of chlorophyll, P, Ca, and Mg. In the case of the K content, only the combined factors had an effect, whereas, for the Na content, the K dose and the combined factors had an effect. Although there was not a clear effect of the treatments on the K plant content, it was remarkable that the biofertilized treatment with no K addition presented a significantly higher K concentration in comparison with the treatment without inoculation. This may imply the action of the rhizobacteria to improve the availability of the non-soluble K present through irrigation and in the cultivation soil, considering that the water was characterized as very hard water, which implies a high content of mineral salts. In this regard, in our previous work [9] on the biofertilization of tomatillo seedlings without the addition of chemical fertilizers, we reported that the rhizobacterial strain *A. calcoaceticus* caused an increase in the K content of the plants in comparison with the non-inoculated treatment. The reason that this effect was observed only in the low K dose seems to be related to the availability of this element. It appears that when K is readily available, the solubilization activity of the inoculant is not needed, so no differences in the plant’s K content are presented in the rest of the treatments. In the case of phosphorous, a similar effect of the inoculant was observed, with an increase in the P content at low K doses. A similar rationale to that applied previously for the biofertilizer effect on K can be further supported considering that in our previous work, the strain *A. calcoaceticus* UTMR2 displayed in vitro capacity for P solubilization [9].

On the other hand, there was not observed a clear effect of the rhizobacterial inoculant on the plant contents of the elements Ca, Mg, and Mn. However, the K fertilization dose influenced the content of these minerals since the treatments with no K fertilization resulted in the highest mineral contents, whereas the opposite effect was observed in the fully fertilized plants. Since this effect was not related to rhizobacterial inoculation, it can be due to competition between K^+^ with both Ca^+2^ and Mg^+2^ ions for binding sites in the soil, which reduces the availability of the latter elements for the plant when K^+^ is in large concentrations in the high K doses (75 and 100%) [22].

In the case of Na content, in contrast to the non-inoculated treatments, the presence of the biofertilizer had a remarkable effect on this element on the plant, with a linear dependence on the K dose (r = 0.93), in which the inoculant appeared to modulate the Na content in the plant as a function of the K concentration in the irrigation water. From the analysis of these components, it can be seen that the soil has a medium level of Na, while the water has a very high amount of Na (7.5 meq/L), which surpasses the recommended limit of 5 meq/L [23]. Excessive amounts of Na^+^ in the soil or in the irrigation water can be detrimental and even toxic to the plant, affecting its growth and development. On the other hand, while K^+^ is an essential cation required by plants in large amounts, which is involved in a great number of biological functions related to a plant’s growth and development, Na^+^ can also help K^+^ as osmoticum and regulate the ionic homeostasis in plants through different families of ion channels and transporters [24]. Therefore, it can be concluded that the rhizobacterial strain regulated the accumulation and transport of K and P in the plant, which was apparently directed to reduce Na toxicity but also to the appropriate utilization of this element to keep the ionic homeostasis in the plant.

In another study, Ali et al. [4] reported that the concentrations of N, P, and K in potato leaves were affected by the *Bacillus cereus* inoculation, with the inoculant increasing the concentration of N, P, and K by 34, 32, and 62%, respectively, in comparison with the non-inoculated plants. In contrast, Cisternas et al. [25] reported that *B. amyloliquefaciens* caused an increase in the Ca content in green bell peppers; however, an opposite effect was observed in the content of K, in which the values of the non-inoculated treatments were higher than those of the inoculated plants.

### 3.2. Effect on Plant Physiology

An increase in chlorophyll content was observed in the inoculated plants at K doses of 75 and 50% in comparison with the rest of the treatments. In this accord, Suzuki et al. [26] reported that an *A. calcoaceticus* strain caused an increase in the chlorophyll content in *Lactuca sativa* plants subjected to low-nutrient conditions. In a similar work [27], the inoculation of the strain *A. calcoaceticus* reduced chlorophyll losses in plants cultivated under drought stress conditions by inducing chlorophyll synthesis at concentrations similar to those of non-stressed cultivated plants. In another study, Foughalia et al. [13] reported that the strains *A. calcoaceticus* and *Bacillus safensis* protected tomato plants against the pathogen *Botrytis cinerea* through the activation of plant defense mechanisms, which additionally caused an increase in the chlorophyll content and growth of the plant from 38.1 to 45.0 SPAD units with the inoculation of *A. calcoaceticus* compared with the non-inoculated control.

The chlorophyll molecule contains Mg, which is bonded to a chlorin ligand. Up to 10% of Mg in plants is associated with chlorophyll synthesis [28]. Considering the fact that the rhizobacterial inoculant increased the Mg content in the treatments with no K fertilization, the increase in chlorophyll content at low K doses may be related to the improved availability and uptake of Mg caused by the effect of the inoculation of *Acinetobacter calcoaceticus* UTMR2 strain. In this regard, Reis et al. [29] reported that the microbial inoculation of *Paenibacillus alvei* and *Bacillus cereus* had a positive effect on biomass accumulation in *Glycine max*, whereas the non-inoculated plants accumulated fewer nutrients and presented reduced chlorophyll index and low photosynthetic rates. Kalaji et al. [30] reported that chlorophyll synthesis is influenced by the content of minerals in the plant, mainly phosphorus, and magnesium; therefore, the deficiency of these elements causes a decrease in the plant concentration of chlorophyll [28]. Considering this, the highest P concentrations obtained at low K doses (KB50 and KB0) with the application of the rhizobacterial inoculant appear to have induced larger chlorophyll contents in these treatments.

### 3.3. Plant Growth Parameters

In general, plants inoculated with the strain *A. calcoaceticus* presented the highest dry weights of tomatillo plants at low doses of K fertilization (50 and 0%). The positive effect on the dry weight of bacterial inoculation was observed for all treatments below the full K dose with respect to the non-inoculated plants. Similar results were found in our previous work on the biofertilization of tomatillo seedlings [9], in which the rhizobacterial strains *Atlantibacter* sp., *Priestia megaterium,* and *A. calcoaceticus* increased the dry leaf weight (>349%), weight (>479%), and plant height (>140%) of tomatillo seedlings compared with the non-inoculated control.

Plant growth-promoting rhizobacteria are capable of improving the availability and absorption of nutrients and water, consequently increasing biomass and productivity in plants [31]. In our study, this was reflected in the fact that the effect of the inoculant on the contents of K and P was more evident in the treatments with low K doses (0 and 50%). There are some reports in the literature regarding the benefits of *A. calcoaceticus* as a biofertilizer. Rojas-Solis et al. [7] reported that the single and combined application of the strains *Pseudomonas fluorescens* and *B. thuringiensis* resulted in a beneficial effect on the development and agronomic parameters of tomatillo seedlings in comparison with the non-inoculated control.

It has been reported that the chlorophyll content of the plant is related to plant-growth improvement in biofertilization experiments [26]. In the present work, it was found that the inoculated treatment at 50% of the K dose with the higher chlorophyll contents presented the largest growth among the treatments in terms of dry weight and plant height, which is probably due to the fact that chlorophyll is an important factor for photosynthesis and plant growth. In this regard, Helaly et al. [32] reported that the improvement in the growth parameters of kale plants (*Brassica oleracea*) inoculated with *Pseudomonas koreensis*, *Ralstonia pickettii*, and *B. cereus* was due to an improved photosynthetic activity as a result of the higher chlorophyll content in the plant leaves.

### 3.4. Fruit Yield

The inoculated treatment at 75% of the recommended K dose (KB75) presented yields that were even higher or comparable with the highest yields obtained in the non-inoculated treatments with a full K dose. However, a similar behavior was observed for the non-inoculated treatment at the same K dose, so it appears that the yield results were due to the effect of the K fertilization dose rather than the application of the inoculant. On the other hand, the inoculated treatments without K fertilization resulted in yield values comparable with the highest obtained values for the rest of the treatments. This may imply that the application of the inoculant had a greater effect at low K doses, in which the bacteria were more active toward plant biofertilization. These results are in accord with the observations made above for mineral content In the biofertilized plants at low K rates.

As stated before, no biofertilization studies have been reported at the fruiting stage of tomatillo crops. In a tomato biofertilization study, Yagmur and Gunes [15] reported that *Bacillus megaterium* increased the fruit yield by 20% in comparison with the control. In addition, similarly to our observations, the rhizobacteria applied in the work (*B. megaterium*, *Paenibacillus polymxa*, *Burkholderia cepacia,* and *Azospirillum* sp.) did not have a significant effect on the fruit size and weight. In an analogous study, He et al. [33] reported that the individual application of the strain *B. pumilus* resulted in higher fruit yields than the non-inoculated control (38.8%). On the other hand, Zapata-Sifuentes et al. [34] reported that *A. radioresistens* and *Sinorhizobium meliloti* increased the dry biomass, size, and yield of cucumber fruits in comparison with the non-inoculated plants.

Apart from enhancing nutrient assimilation by the plant, benefic rhizobacteria can favor plant growth by synthesizing phytohormones, such as indole-3-acetic acid (IAA). Although the presence of these metabolites in the plants was not studied in this work, in our previous publication [9], it was found that the strain *A. calcoaceticus* UTMR2 was capable of in vitro producing IAA (4.5 µg/mL), with the inoculant inducing a higher growth of tomatillo seedlings in comparison with the non-inoculated control. A few studies have reported the presence and effects of phytohormones during in vivo crop cultivation. In a pepper biocontrol study, Abdelaziz et al. [35] reported that the application of cyanobacteria during cultivation increased the growth and enhanced salicylic acid (SA) and IAA in the *Fusarium* sp. infected plants while decreasing abscisic acid (ABA). In a similar study, Singh et al. [36] reported that the inoculation of cyanobacteria increased the accumulation of the phytohormones IAA and indole butyric acid in rice leaves.

### 3.5. Fruit Nutritional Quality

There are no reports in the literature regarding the effect of rhizobacteria on the quality of the tomatillo fruit. In a tomato biofertilization study, Yagmur and Gunes [15] reported that the single use of the strains *B. megaterium*, *Paenibacillus polymxa*, *Burkholderia cepacia,* and *Azospirillum* sp., increased the TSS, the electric conductivity, and the pH of tomato fruits. In a similar work, Gashash et al. [37] reported that the co-inoculation of the strains *B. subtilis* and *B. amyloliquefaciens* produced a beneficial effect on the yield and quality parameters of tomato fruits with respect to the non-inoculated control: the number of fruits per plant (76%), fruit weight (36%), fruit size (50%), ascorbic acid (75%), and in the contents of N, P, and K in the fruit. Additionally, in a work on the inoculation of *B. amyloliquefaciens* in pepper cultivation [25], the inoculant applied in the seedbed before transplant resulted in higher contents of protein, fat, Ca, and Fe with respect to the non-inoculated control. In addition, the authors reported that the bacterial strain did not have an effect on carbohydrate and ash contents.

From the ANOVA assessment (Table S4), both factors (inoculant and K dose) had a significant effect, both individual and combined, on the most important parameters of the fruit: yield indexes, FRAP antioxidant activity, and the contents of protein, fiber, and fat. Only in the case of the yield index no significant individual effect of the inoculant was observed, whereas a significant effect was determined for the combination of this factor and the K dose (Table S4). The results obtained in this work for TSS were similar to those reported in the literature for *P. ixocarpa*, which ranged from 5.1 to 6.5 °Brix [38]. From the TSS results, it can be inferred that the rhizobacterial inoculant had a modulating effect on the content of sugars in the fruit, in which the sugar content increased with the dose of K fertilization (r = 0.996). The values of titrable acidity of tomatillo in this study are comparable with those reported in the literature, which range from 0.7 to 1.7% [39]. The pH of the fruits was also within the values reported elsewhere, which ranged from 3.7 to 4.4 [40,41].

The interaction between the total soluble solids and the acidity of the products produces taste and flavor, which are highly dependent on the maturity of the fruit. The sugar:acidity ratio has been used mainly in studies of tomato post-harvest analysis related to fruit maturity and taste [42,43]. A few works in the literature report this parameter for tomatillo fruits. The sugar:acidity ratio obtained in this work was in agreement with other reports in the literature for tomatilloes. Ostrzycka et al. [40] reported a ratio from 6.3 to 11.0 for tomatillo fruits var. Rendidora. Curi et al. [44] reported a very low MI value (2.6) for *P. ixocarpa* and slightly low values for other *Physalis* species (4.9–5.6). Pérez-Herrera et al. [39] reported MI values for wild *Physalis* spp. genotypes, which ranged from 3.6 to 8.8. Considering this, it can be stated that, in general, the MI of the fruits produced in this study is moderately high according to the results reported in the literature.

A high MI value indicates a greater rate of carbohydrate hydrolysis in the fruit and consumption of organic acids during fruit respiration, with a consequent reduction in fruit acidity. This index has been used to assess the shelf life of tomato fruits during post-harvest processes in which a high MI value implies a shorter shelf-life of the fruit product [42,43]. Despite the fact that no clear pattern was observed in the MI values for the different treatments, the biofertilized treatment with a 75% K dose (KB75) and the treatment with no K addition without biofertilization (KF0) had the lowest MI values, which may indicate that the product has a larger shelf life compared with treatments with high MI values. However, careful consideration of this index is necessary since not only is fruit maturity important to assess the quality of the fruit product, but also the nutritional parameters need to be taken into consideration. In this regard, no considerable difference was observed for these treatments in terms of the nutrimental components, which showed dependence on the K dose or the biofertilizer inoculation (protein and fiber), so it appears that the low maturity of the fruits in these treatments did not negatively affect their nutritional quality.

The protein contents obtained in this work for the tomatillo fruits were similar or superior to those reported in the literature [1,39,41,45], with values between 0.65 and 1.18%. In terms of the treatments performed in this work, at moderate or high K doses (50, 75, and 100%), it appears that the fruit content is related to a K dose threshold (50% of the K dose), above which the protein concentration is significantly higher. This effect was more pronounced in the biofertilized treatments, in which an abrupt boost was observed when passing from low (0–50%) to high (75–100%) K doses. On the other hand, in general, there is not a clear effect of the rhizobacterial inoculant on the protein content. However, in the non-biofertilized treatment with a null K addition, the protein content of the inoculated treatment (0.6%) was considerably lower than its counterpart without inoculation (1.4%), which indicates that the bacterial inoculation had a detrimental effect on the protein content of the fruit at very low K inputs. These results agree with the results reported by Ochoa-Velasco [18] in the biofertilization of tomato with a *B. licheniformis* strain, in which lower protein concentrations were achieved in the inoculated treatments in comparison with their counterparts without inoculation at different N fertilization doses. This effect was possibly attributed to the high nutrient demands by the colonizing bacteria, which affected protein biosynthesis or the modulating mechanism for homeostasis in the fruit cells.

In general, the fat content in tomatillo fruits was lower than that reported in the literature, which ranged from 1.0 to 2.1% [1,41,45]. Similar to the protein results, in the rhizobacterial strain, low K doses (0 and 50%) produced considerably lower fat contents than those observed at high K doses (75 and 100%). This seems to corroborate the hypothesis that the rhizobacterial strain modulates the biosynthesis of macromolecules (proteins and lipids) at low K doses. In terms of fiber, the results of this study are in agreement with those mentioned in the literature, which range from 1.5 to 4.3% [39,45]. The rhizobacterial inoculant had a positive effect on fiber biosynthesis in the fruit at low K doses, particularly at a K dose of 50%. In the case of the biofertilized treatments at low K doses, high fiber contents are inversely related to the contents of total soluble solids, protein, and fat. These results seem to support the bacterial regulation effect in the synthesis of macromolecules at low K doses, which may involve the production of non-hydrolizable carbohydrate molecules that can serve as an energy reserve for the fruit during its maduration and senescence. Particularly in terms of carbohydrates, as stated above, it was evident that the metabolic modulation included the increase of sugars in the fruit with the amount of K in the irrigation water, which at low K doses resulted in a reduction in the sugar content and an increase in complex carbohydrates that can play a role as reserve carbohydrates in the fruit. In contrast, in the tomato biofertilization study reported above [18], the *B. licheniformis* inoculant at 50 and 75% N doses presented the highest fiber contents among the treatments, which were higher than their non-inoculated counterparts; while lower N doses resulted in reduced fiber contents regardless rhizobacterial inoculation.

The mineral content in tomatillo fruits is an important attribute that determines the nutritional quality of the fruits. The range of the mineral content values for the treatments was in agreement with that reported by Bock (0.8–1.4%) [41]. From the biofertilization experiment, it was observed that neither the K dose nor the application of the inoculant had an effect on the ash content. An analogous behavior was observed as regards the electric conductivity, a result that was expected since this parameter is related to the number of mineral ions in the sample. Similar results were obtained in a study on tomato biofertilization [18] in which the ash content was not affected by the *B. licheniformis* or the nitrogen applied to tomato plants. Likewise, Katsenios [17] reported that the pH, as well as the ash content, of the tomato fruits were not significantly affected by the application of inoculants belonging to the *Bacillus* genus.

Regarding the contents of mineral elements in tomatillo fruits, the values obtained in this work were similar to those reported in the literature. Comparing our results with those for *P. ixocarpa* var. Rendidora [40], the authors reported lower contents of Na (0.29 g/kg) and Mg (1.1 g/kg) and higher of K (32.5 g/kg fruit) and Ca (0.31 g/kg). In addition, the content of P in the tomatillo fruits in this work was considerably larger than the value reported by Cardenas-Castro [45] (0.39 g/kg), while the Ca content was comparable to that reported elsewhere (0.07 g/kg) [46]. On the other hand, no studies of the effect of rhizobacterial inoculants on the mineral content of tomatillo fruit have been reported. In a study of tomato biofertilization, He et al. [33] evaluated the single and combined application of the rhizobacterial strains *B. pumilus* and *Pseudomonas putida* on tomato cultivation. The single application of these strains resulted in higher Na (80.5%) and Mn (29.5%) contents as compared with the non-inoculated control by the strains *P. putida* and *B. pumilus*, respectively, with these results representing the highest values of these elements among the treatments. In terms of the macronutrients, the single application of the strain *P. putida* increased the content of P (16.9%) and K (12.9%) in the tomato fruits.

From our results, it can be concluded that neither the biofertilizer application nor the K fertilization dose had a significant effect on the content of the mineral elements tested: K, P, Mg, and Mn, whereas no clear trends were observed for Ca and Na. This seems to indicate that the translocation of these elements is not a limiting factor at the fruiting stage of the plant, which may imply that, in contrast to the plant growth stage, mineral availability is not critical in fruit development. Apparently, for the variables studied in this work (K dose and rhizobacterial inoculation), at the fruiting stage, the plants were well prepared for the biosynthesis of the fruit cell structures. In an asparagus biofertilization study, Xekarfotakis et al. [47] reported that the contents of N, P, and K in the leaves and roots of the plants were similar among the different chemically and biologically fertilized treatments. This result implies that these macronutrients were not limiting the growth and development of the plants. Accordingly, in the present study, the translocation of minerals from the leaves and stem to the fruit appears to be controlled by both nutrient availability and mineral requirements of the fruits. This is noteworthy, considering that the K dose and rhizobacterial inoculation are quite important for the development of tomatillo plants and fruits. In a previous study [9], it was attested that the rhizobacterial inoculant *A. calcoaceticus* UTMR2 could improve the solubility of several mineral elements, making them more accessible to the roots. This effect of the inoculant can reduce the requirements of chemical fertilizer for plant growth and fruit development.

### 3.6. Phenolic Compounds and Antioxidant Activity

The content of total phenolic compounds (TPC) was slightly higher than the values reported by González-Mendoza et al. [48], who indicated contents ranged from 530 to 1080 mg GAE/100 g for different genotypes of tomatillo, whereas lower TPC values (53.0–112.4 mg GAE/100 g) were reported by da Silva et al. [49] for different species of *Physalis*. These authors also pointed out that the variability among the content of bioactive compounds depends on several factors, including the light spectrum. El-Beltagi et al. indicated that the most abundant phenolic compounds presented in *P. peruviana* fruits are gallic acid and its derivatives [50].

Though a few studies have reported the antioxidant capacity of different species of *Physalis*, their results are not comparable with those found in this study due to differences in methodologies and quantification procedures. However, a good correlation (r > 0.77) between total phenolic compounds and FRAP antioxidant capacity was obtained, while no correlation was obtained between phenolic compounds and DPPH assay (0.00) and between the antioxidant assays (−0.36). A similar behavior was observed by other authors for different species of *Physalis*, in which a negative correlation between phenolic compounds and antioxidant capacity (DPPH assay) was observed [44,48]. Interestingly, a positive correlation was reported between phenolic compounds and FRAP in both domesticated and wild fruits of *P. peruviana* [51].

As stated above, no biofertilization studies of tomatilloes have been reported in the literature. Regarding tomato biofertilization, Ochoa-Velasco et al. [18] reported that the application of the strain *B. licheniformis* at 75% of the full N dose increased the antioxidant capacity of the fruits for the DPPH (1.9×) and the FRAP (1.4×) assays with respect to the non-inoculated treatment. Similarly, Ruiz-Cisneros et al. [52] reported an increase in the content of phenols (1.5×) and antioxidant activity (2.8×) in tomato fruits biofertilized with *Bacillus* spp. strains in comparison with the control without inoculation. In this work, the significant effects of the K fertilizer and beneficial rhizobacteria were observed only for FRAP antioxidant capacity. In the treatment without inoculation, the FRAP antioxidant capacity of tomatillo fruits increased with the K dose. However, a contrary effect was observed when the rhizobacterial inoculant was added, obtaining the highest antioxidant values in the treatment without the addition of K fertilization (KB0). This effect is evident when comparing the antioxidant capacity between non-K-fertilized treatments with and without rhizobacterial inoculation (1891.08 and 1170.17 mg AA/100 g, respectively), representing a 61.6% increase. Therefore, it can be inferred that the use of the rhizobacterial Inoculant can reduce the environmental stress caused by the low nutrient available by increasing the biosynthesis of bioactive compounds with an antioxidant capacity [53].

### 3.7. PCA Multivariate Analysis

From the PCA and heatmap study on the main parameters that influenced the nutritional, yield, and bioactive properties of the tomatillo fruits, it can be inferred that both the K dose and the presence of the rhizobacterial inoculant had a significant influence on the overall characteristics of the plants and fruits for the different treatments. To the best of our knowledge, this is the first study that assessed fruit and plant parameters with fertilization and rhizobacterial inoculation regimes in a multivariate study. According to the analysis, three clusters were detected in which the following treatments were grouped: T4 and T5; T6 and T1; and T3 and T2 (Figure 2). From the PCA biplot and heatmap, the following plant and fruit parameters were grouped: (i) Na plant concentration and fruit protein; (ii) plant dry weight and fruit fiber; (iii) FRAP antioxidant capacity and yield index; and (iv) plant content of Mg and Ca.

**Figure 4:**
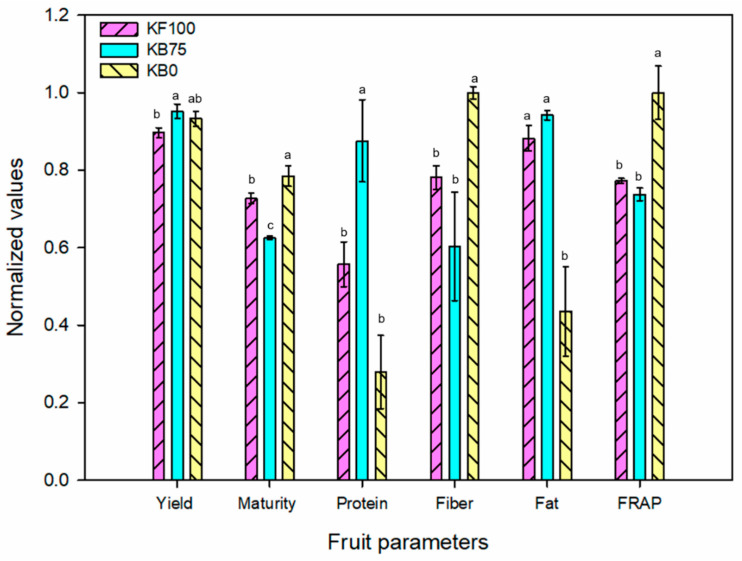
Comparison of the normalized main parameters indicating the quality of tomatillo fruits (*Physalis ixocarpa* Brot. cv. “cáscara morada”) in the evaluation of the biofertilizing effect of *Acinetobacter calcoaceticus* UTMR2 for selected treatments at different doses of potassium. KF#: treatments with chemical fertilization only; KB#: treatments with chemical fertilization and biofertilization; #: potassium fertilization dose (%) based on the recommended dose for tomatillo cultivation [20]. Data normalization and mean comparisons were calculated considering all the treatments of the study. Different letters in the columns for each parameter indicate a significant difference among the treatments according to the HSD Tukey test (*p* < 0.05, n = 3).

Taking this and the heatmap analysis into consideration, the fully fertilized treatment without fertilization (KF100), which had the ideal fertilization dose, was located close to the center of the biplot graph. In addition, the biofertilized treatment at the 75% dose (KB75) was the closest treatment in the PCA graph. This is attested to in the heatmap, in which both treatments are associated in the dendrogram. This result is significant as it indicates that the biofertilized treatment with a 25% reduced dose in K fertilization achieved a similar output in the plant and fruit parameters to the fully fertilized treatment without rhizobacterial inoculation. To better visualize this, the normalized data of the main parameters for fruit quality for the treatments KF100 and KB75 were plotted (Figure 4). As can be observed, the values for the biofertilized treatment (KB75) were comparable and even greater than those for the non-inoculated fully fertilized treatment in terms of total yield index and protein. This confirms the observations of the multivariate analysis.

Perhaps the most remarkable effect of the biofertilizing activity of the strain *A. calcoaceticus* UTMR2 was observed at low K doses (0 and 50%); treatments, which were clearly separated in the dendrogram of the heatmap in a different group from the corresponding non-inoculated treatments. Outstandingly, the biofertilized treatment with null K fertilization (KB0) resulted in the highest values for most of the plant and fruit parameters. The fruit’s yield and quality parameters of this treatment and the non-inoculated fully-fertilized samples (KF100) were compared in Figure 4. While the yield and protein values were comparable in both treatments, the KB0 treatment resulted in higher fiber content and FRAP antioxidant capacity, and lower fat content, than the KF100 treatment. These results clearly indicate the modulating effect of the biofertilizer in the growth and fruit production of tomatillo plants, which seems to be related to the increase in the assimilation of nutrients from the soil and water supply. In order to thoroughly assess the quality of the fruit products without K fertilization, it would be interesting to investigate the effect of biofertilization on other bioactive compounds in the fruit, such as the content of vitamin C, carotenes, and lipid profile.

### 3.8. Metabolomic Analyses

An untargeted metabolomic analysis was performed for the samples treated with a 75% K dose (biofertilized and non-biofertilized). From the analysis of the metabolomic heatmap (Figure 3a), it was concluded that the inoculant *A. calcoaceticus* UTMR2 had a large effect on the global metabolic reactions of the plant, in which a decrease in the content of most of the detected metabolites prevailed. These results agreed with the chemical profiling analysis, in which only 7 compounds were over-accumulated in the biofertilized sample, in contrast with over 200 compounds, which were favored in the non-inoculated treatment. So it can be concluded that the rhizobacterial inoculant modulates the metabolic reactions in tomatillo fruits through the promotion of the biosynthesis of only a few metabolites, which should possess important biochemical activity. In contrast, in a metabolomic study on tomato biofertilization with the inoculation of *Pseudomonas aeruginosa* [19], the authors reported that the presence of the bacterial inoculant altered the metabolic pathways in the fruits by the up-regulation of a wide variety of metabolites belonging to sugars, alcohols, alkaloids, terpenoids, flavonoids, carotenoids, and organic acids. In addition, the inoculant induced the production of carotenone, cycloartanol, and amino chlorocoumarin.

On the other hand, as stated above, from the PCA analyses, considering the treatments at 75% K dose, the addition of the biofertilizer resulted in plant and fruit attributes similar to the fully fertilized dose without rhizobacterial inoculation, a treatment that can be considered as the ideal positive control. Moreover, in the heatmap dendrogram, this treatment was separately grouped from the non-inoculated treatment at the same K dose, which again demonstrates the significant effect of the rhizobacteria on the yield, as well as on the nutritional and antioxidant properties of the tomatillo fruits.

Two of the main metabolites that were putatively identified in the untargeted metabolomic analysis were 4,4″-bis(N-feruloyl)serotonin and salvianolic acid K, a carboxylic acid polyphenol with fold-change values of 15.4 and 8.0, respectively. The compound 4,4″-bis(N-feruloyl)serotonin is a hydroxycinnamate amide (HCAA). Interestingly, different HCAAs have been identified as chemical markers in plant-pathogen and plant-insect (e.g., herbivore) interactions, suggesting their involvement in the defense response [54]. Therefore, the accumulation of this metabolite may be part of the biochemical response to the interaction of tomatillo plants and *A. calcoaceticus* UTMR2. A number of biological functions have been reported for serotonin derivatives, which include an antioxidant, antityrosinase, inhibitor of melanin production, antitumor, and inducer of fibroblast growth [55]. Very few works in the literature have reported the biological functions of this compound in plants: biosynthesis of serotonin was highly induced upon senescence in rice plants, indicating that it may be associated with an aging-inhibitory mechanism because of their strong antioxidant activity [56]. On the other hand, Kosović et al. [57] reported the extraction of the serotonin derivative from the Maral root (*Rhaponticum carthamoides*), which is an herb that has been used in alternative medicine as a toning agent because of its effects on strengthening the nervous system and favoring mental health.

On the other hand, salvianolic acid K has been identified from *Salvia bulleyana* Diels [58], a Chinese endemic plant, as the majority compound in the roots of the plant (12.3 mg/g dw). The roots of this plant have been used in traditional Chinese medicine to alleviate emotional and sleeping disorders as well as to treat some diseases, such as coronary heart disease, as well as liver and kidney ailments [59]. Although, to date, the activity of this acid in plants has not been reported in the literature, it is possible that this compound has antioxidant activity in plants against biotic and abiotic stresses, similar to its analogous compound salvianolic acid B, identified in the plant *S. miltiorhiza*. Salvianolic acids are potent antioxidants that have the capacity to reduce intracellular as well as intravascular oxidative stress, thus protecting endothelial cells and aortic smooth muscle cells and preventing LDL from free radical damage and peroxidation. In addition, these compounds have an indirect function in the regulation of the cell’s immune systems [60].

Considering that phenolics are one of the most known bioactive compounds, and some of them were tentatively identified in the untargeted metabolomics approach, we performed a phenolics-targeted metabolomic analysis. From this analysis, chlorogenic acid was the main phenolic compound identified that was produced in larger amounts in the inoculated treatment in comparison with the non-biofertilized control. This acid can be a precursor for the synthesis of more complex phenolic compounds since the chlorogenic acid pathway is one of the main routes for polyphenol biosynthesis. Chlorogenic acid, the major polyphenolic compound present in potatoes, has been related to the enhancement of plant disease resistance through the inhibition of plant pathogens [61]. In another study, Wojciechowska et al. [62] reported that chlorogenic acid reduced the infection of the phytopathogenic fungi *Alternaria alternata* of tomato fruit by inhibiting the synthesis of the toxin alternariol, which promotes fungal colonization of the plant. The authors confirmed that chlorogenic acid plays an important role in the plant defense system of the plant by performing a metabolomics analysis.

## 4. Conclusions

In general, the application of the inoculant *Acinetobacter calcoaceticus* UTMR2 positively affected several parameters of both plant growth and the fruit nutritional quality of tomatillo crops (*Physalis ixocarpa* Brot.) cultivated at reduced doses of potassium. The main biofertilization mechanism was thought to be the bacterial inoculant favoring nutrient assimilation by the plant, but phytohormone and the synthesis of growth-promotion factors should not be discarded. By means of a multivariate analysis using PCA and heatmapping, it was concluded that the inoculant induced a strong modulating activity in tomatillo plants in the evaluated parameters, with a remarkable effect at low K doses (0 and 50%). The inoculated treatment at 75% of the K dose resulted in similar plant and fruit characteristics to the fully fertilized control, which is remarkable since this represents a 25% reduction of the full K dose. On the other hand, the biofertilized treatment with no K addition resulted in the highest values in the plant and fruit parameters, except for the contents of protein and fat. Further studies would be required to attest to the overall nutritional and functional quality of these fruits.

In addition, from the metabolomics analysis of the fruits at 75% of the K dose, a remarkable modulating effect of the rhizobacteria was observed by the up-regulation of a few key metabolites, i.e., feruloyl serotonin, salvianolic acid, and chlorogenic acid, which may have a role in anti-senescence and resistance mechanisms. In addition to metabolomics analysis, a proteomic and transcriptomic approach would be crucial to improve the understanding of the synthesis of bioactive compounds in the fruit. In addition, a functional genomic analysis from the whole genome of the *A. calcoaceticus* strain would be desirable to further elucidate both plant growth promotion and modulation of the biochemical mechanisms in the fruit. In conclusion, the rhizobacterial strain had a positive effect on plant growth, nutritional quality, bioactive compounds, and antioxidant activity of tomatillo fruits at reduced doses of K fertilizer, which gives support for its consideration as an effective biofertilizer strain.

## 5. Materials and methods

### 5.1. Experimental Site and Design

To perform the agronomical experiment, a macro tunnel was established at the village of San José de La Pradera located in the municipality of Santa Cruz Tacache de Mina, Oaxaca, México (17°47′5″ North and 98°9′2″ West at 1112 m.a.s.l.). In this study, certified tomatillo seeds of *Physalis ixocarpa* Brot. cv. “cáscara morada” were used as the crop cultivar. An analysis of the water and soil used in this experiment was performed (NOM-021-RECNAT-2000) [21]. The soil texture was loamy-clayey-sandy with an organic matter content of 2.0%, pH of 8.0, and electrical conductivity of 1.4 dS/m. The concentration of macronutrients in soil was the following: 202, 261, 3857, 332, 58.1, 57.0, and 107 ppm for P, K, Ca, Mg, NO_3_, S, and Na, respectively, and micronutrient concentrations of 4.67, 0.78, 3.32, 2.90 y 0.85 ppm for Fe, Zn, Mn, Cu, and B, respectively. The pH of the water was 7.4, and electrical conductivity was 1.7 dS/m with a hardness of 41.6. The content of the macronutrients was 4.30, 4.06, 7.54, 0.37, 4.02, and 0.61 meq/L of Ca, Mg, Na, K, SO_4_, and NO_3_, respectively, and of micronutrients 1.04, 0.17, 0.18, 0.001, and 0.002 ppm of B, Fe, Mn, Cu, and Zn, respectively.

The experiment followed a completely randomized design with 8 treatments, which included chemical-only or rhizobacterial-aided fertilization with different potassium concentrations (Table 7). Four K doses (0, 50, 75, and 100%) were used as related to the recommended fertilization dose using Steiner’s universal nutrient solution at 20% [20] and using KNO_3_ as the only source of potassium. A concentrated solution (5 L), adjusted to a pH from 5.5 to 6.5 with nitric acid, was used to prepare 250 L of nutrient solution for irrigation. The irrigation regime was based on the requirements of the plant according to the stage of development. Three irrigations were made daily, in which 100 mL per plant was applied at transplantation and 300 mL during the fruiting stage.

Planting was conducted in 200-cavity polystyrene trays. A mixture of peat moss and perlite (3:1 *v*/*v*) was used as a substrate and remained for 30 days. During this period, the plants were fertigated daily with a Stenier nutrient solution at 20% of the concentration [20]. Tomatillo seedlings were transplanted on 15 August 2021 and harvested three times during the production cycle at 44, 54, and 70 days after transplanting (DAT). Plants were grown in plastic bags of 18 kg with a mix of site soil and river sand (60:40 *v*/*v*); disinfection was conducted by solarization, placing the soil 20 cm thick onto a plastic mantle for 45 d. The space between rows and between plants in the row was 70 cm.

The mean, maximum, and minimum daily temperatures and relative humidities were measured with a HOBO Pro v2 temp/RH meter. The weather parameters were monitored during the experimental period. The average relative humidity was 71%, with a maximum and minimum of 99 and 19%, respectively, and a mean temperature of 25 °C, with maximum and minimum average temperatures of 46 and 12 °C. The photosynthetically active radiation (PAR) was measured with a Light Scout^®^ Mod. 3415FSE quantum light meter (Spectrum Technology Inc. USA) between 10:00 and 16:00 h, with an average PAR measurement of 997 µmol/m^2^/s. For the experiment, 3 replicates were used per treatment, with every experimental replicate consisting of 7 tomatillo plants.

### 5.2. Rhizobacterial Inoculant

The rhizobacteria *Acinetobacter calcoaceticus* UTMR2 was used as the microbial inoculant in this study. The bacterial strain was isolated and characterized in a previous study for its biofertilizing traits on tomatillo seedlings [9]. The inoculant was first tested for optimal growth. After that, the strain was cultured in LB broth and incubated at 37 °C for 72 h in an orbital shaker at 150 rpm to obtain an optical density (600 nm) of 0.2. The concentration of the bacterial suspension was adjusted to 1 × 109 UFC/mL, and then 10 mL of the inoculant was added to the tomato plants. The inoculants were applied twice at 10 and 25 DAT.

### 5.3. Agronomic Parameters

Physiological measurements of the plant were conducted. Photosynthesis rate was measured 60 days after transplant (DAT) with a photosynthesis-fluorescens-meter LI-6400XT (LI-COR, USA) on fully expanded leaves when the sky was clear. Similarly, the chlorophyll measurements were performed at 60 DAT. Six plants of each treatment were selected randomly, after which 2 leaves per plant were measured 3 times. The chlorophyll measurements were performed with a FieldScout CM 1000 chlorophyll meter (Spectrum, USA). The height and stem thickness of the plants were measured at 70 DAT when the experiment was finished. In addition, the dry weight (g/plant) was determined in an analytical scale after the samples were oven-dried at 70 °C for 72 h. The yield of tomatillo fruits (g per plant) was obtained. All tomatillo fruits were counted and weighed. An overall fruit yield index was considered by averaging the normalized fruit yield parameters: fruit weight per plant, number of fruits per plant, and fruit weight. The radial and equatorial diameters of the fruits were measured with a digital caliper.

### 5.4. Physicochemical Parameters of Tomatillo Fruits

The fruits were harvested when it was observed that the fruit completely filled the calyx at 70 DAT. Then, the fruits were removed from the calyx. The CIELab color parameters of tomatillo fruits were determined with an UltraScan Vis Spectrophotometer (HunterLab, USA). For phytochemical analyses, the fruits of all samples were cut in halves and subsequently blended in a home blender for 60 s. The samples were stored in high-density plastic bottles at −40 °C for further analysis. Total soluble solids (°Brix) were determined using an MA871 digital refractometer (Milwaukee, USA). In addition, the pH and electrical conductivity (CE) of the fruit juice were measured with a digital meter (Hanna, USA). A proximal analysis of the fresh samples was performed according to the AOAC methods [63] for the following determinations: moisture, protein, ash, total fiber, and titratable acidity (TA). To perform fat and fiber determinations, the samples were first oven-dried at 105 °C for 3 h.

### 5.5. Fruit Extraction for Bioactive and Metabolomic Analyses

To prepare the fruit samples for phenolic and antioxidant activity assays, the samples were dried at 60 °C until they had constant weight. The dried samples (1 g) were placed in 25 mL of distilled water for extraction with agitation in a hotplate stirrer at room temperature for 1 h. After the extraction time, the sample was cotton-filtered and immediately used for phenol quantification and antioxidant assays.

For metabolomics analysis, a subsample of 35 g was taken from the pooled fruit samples for a selected K fertilization condition (with and without rhizobacterial inoculation). The sample was first frozen at −40 °C in a deep freezer for 48 h, followed by lyophilization in a 75,200 freeze-dryer (Labconco, USA) until the equilibrium moisture was achieved. The dehydrated samples were stored in dry conditions at 4 °C until further analysis. Methanolic extracts were prepared with a Dionex ASE 350 accelerated solvent extraction system (Dionex, USA) using 0.5 g of dried samples. The oven temperature was 60 °C, and two static cycles of 5 min were used. The extracts were concentrated to dryness in an RII rotatory evaporator (Büchi, Switzerland). Dried methanolic extracts were dissolved in methanol (50 mg/mL), filtered with 0.5 μm PTFE membranes, and placed in 2 mL UPLC vials.

### 5.6. Total Phenolic Content and Antioxidant Capacity

Total phenolic compounds were evaluated according to the methodology proposed by Hernández-Carranza et al. [64]. In brief, 1 mL of extract was mixed with 1 mL of the Folin-Ciocalteau reagent (0.1 M) for 3 min; subsequently, 1 mL of Na_2_CO_3_ solution (0.05% *w*/*v*) was added to the mixture. After 30 min at room temperature, the mixture was read at 765 nm using a 6405 UV-vis spectrophotometer (Jenway, UK). Total phenolic compounds were quantified using a standard curve of gallic acid (R^2^ = 0.980).). The results were expressed as mg of gallic acid equivalent (GAE) per 100 g dw.

The antioxidant capacity was evaluated by two different methodologies: DPPH radical inhibition and the ferric reducing antioxidant power (FRAP) assays. The DPPH antioxidant activity was evaluated according to the methodology reported by Hernández-Carranza et al. [64]. One mL of the extract was mixed with 1 mL of DPPH radical solution (0.004% *w*/*v*). The mixture was left in repose for 30 min at room temperature in the dark, and then it was read at 517 nm using a UV-vis spectrophotometer. A standard curve of Trolox (R^2^ = 0.991) was used to calculate the antioxidant capacity. In addition, the FRAP assay was conducted following the methodology reported by Dorman et al. [65]. One mL of the extract was mixed with 2.5 mL of phosphate buffer (0.2 M, pH 7) with 2.5 mL of a C_6_FeK_4_N_6_ solution (1% *w*/*v*) and incubated for 30 min at 50 °C; subsequently, 2.5 mL of a C_2_HCl_3_O_2_ solution (10% *w*/*v*) was added. After that, the mixture was centrifuged for 10 min at 1000 rpm. Finally, 2.5 mL of the supernatant was mixed with 0.5 mL of a FeCl_3_ solution (0.1% *w*/*v*) and 2.5 mL of distilled water. The absorbance was read at 700 nm using a UV-vis spectrophotometer. The results were expressed as mg of ascorbic acid (AA)/100 g of a sample using a standard curve of ascorbic acid (R^2^ = 0.988).

### 5.7. Metabolomic Analyses of Fruit Extracts

#### 5.7.1. Untargeted Metabolomics

The chromatographic system was a Class I UPLC (Waters, USA) coupled to a quadrupole-time of flight Synapt G2-Si mass spectrometer (Waters, USA). The chromatography was conducted on a Waters Acquity BEH column (1.7 μm, 2.1 × 50 mm) with temperatures of 40 and 15 °C for the column and the sample, respectively. The mobile phase consisted of (A) water and (B) acetonitrile, both with 0.1% of formic acid (Sigma-Aldrich, USA). The gradient conditions of the mobile phases were: (i) 0–20 min, a 1–99% B linear gradient; (ii) 20–24 min, 99% B isocratic; (iii) 24–25 min, 90–1% B linear gradient. The flow rate was 0.3 mL/min with an injection of 5 µL of the extracted sample. The mass spectrometric analysis was performed with an electrospray ionization source in negative mode with voltages of 3000, 40, and 80 V for the capillary, sampling cone, and source offset, respectively. The temperature of the source was 120 °C. The desolvation gas flow rate was 600 L/h at 20 °C, and the nebulizer pressure was 6.5 bar. Leucine-enkephalin was used as the lock mass (554.2615, [M-H]^−^). The conditions used for MS analysis were: mass range 50–1200 Da, function 1 CE, 6 V, function 2 CER 10–30 V, scan time 0.5 s. The data were acquired and processed with the software MassLynx v. 4.1 and MarkerLynx v. 4.1 (Waters, USA). The tentative identification of metabolites was performed using the MetaboAnalyst bioinformatic platform (https://www.metaboanalyst.ca/MetaboAnalyst/home.xhtml, accessed on 7 September 2022).

#### 5.7.2. Phenolics Targeted Metabolomics

The chromatographic system was a UPLC 1290 infinity (Agilent, USA) coupled to a 6460 triple quadrupole mass spectrometer (Agilent, USA). The chromatography was conducted on an Agilent Eclipse Plus C18 column (1.8 μm, 2.1 × 50 mm) heated at 40 °C. The mobile phase consisted of (A) water and (B) acetonitrile, both with 0.1% of formic acid (Sigma-Aldrich, USA). The gradient conditions of the mobile phases were the following: (i) 0–30 min, 1–50% B a linear gradient; (ii) 30–35 min, a 50–99% B linear gradient; (iii) 35–39 min, 99% B isocratic; (iv) 39–40 min, a 90–1% B linear gradient. The flow rate was 0.3 mL/min with an injection of 2 µL of the extracted sample. The mass spectrometric analysis was performed with an electrospray ionization source in positive and negative modes with gas and sheath gas temperatures of 300 and 250 °C, respectively. The gas and sheath gas flow rates were 5 and 11 L/min, respectively. The nebulizer pressure was 45 psi, and the capillary and nozzle voltages in positive and negative modes were 3500 and 500 V, respectively. Each phenolic compound was identified and quantified by using the dynamic multiple reaction monitoring (dMRM) method. Table S5 describes the chromatographic and spectrometric conditions for each phenolic compound in addition to the quantification range, the type of regression used, and the value of the coefficient of determination. In addition, *t*-tests were performed on the Log_10_ normalized data using the MetaboAnalyst bioinformatic platform (https://www.metaboanalyst.ca/MetaboAnalyst/home.xhtml, accessed on 7 September 2022).

### 5.8. Determination of Mineral Elements

The mineral content (P, K, Ca, Na, Mg, and Mn) of the fruit and plant samples was quantified using inductively coupled plasma with optical emission spectroscopy (ICP-OES) Optima 7000 equipment (Perkin Elmer, USA). For the ICP-OES analysis, the samples were oven-dried at 60 °C for 48 h. Subsequently, the samples were subjected to acid digestion with reflux and later analyzed in a Spectroblue instrument equipped with the Spectro Smart Analyzer software, according to Ramírez-Cariño et al. [9]. Calibration curves for minerals (P, K, Ca, Na, Mg, and Mn) were performed using individual standard solutions (Perkin Elmer, USA). The linear correlation coefficient was 0.9999 for each mineral. Measurement results are presented as mean value ± standard deviation and are expressed as g/kg dw.

### 5.9. Statistical Analysis

A two-way analysis of variance (ANOVA) was performed to determine the effect of *Acinetobacter calcoaceticus* application at different doses of potassium. For the experimental data analysis, the statistical software SAS v.9.00 (SAS Institute Inc., USA) was used. The comparisons of means were made using Tukey’s honestly significant difference test at the 5% level of significance (*p* < 0.05). Similarly, for paired comparisons, a *t*-test was performed (*p* < 0.05). To check the normality of data, the Shapiro-Wilk and Kolmogorov-Smirnov normality tests were performed, considering a 95% confidence level. In addition, a multivariate analysis was conducted by means of a principal component analysis (PCA) and a heatmap to analyze the overall effect of the treatments considered in the study on the most relevant parameters related to tomatillo plants and fruits, in which significant differences among the treatments were observed. A clustering analysis was also performed in this study. The software RStudio (2022.07.2 + 576 “Spotted Wakerobin”) was employed for data analysis using the ggplot2 library and the functions biplot and heatmap for the PCA and heatmap graphs, respectively.

## Figures and Tables

**Table 1:** Mineral/element content in tomatillo plants (g/kg dw) in the evaluation of the biofertilizing effect of *Acinetobacter calcoaceticus* UTMR2 in tomatillo plants (*Physalis ixocarpa* Brot. cv. “cáscara morada”) at different doses of potassium.

Treatment ^1^	K	P	Ca	Na	Mg	Mn
KF100	25.9 ± 4.61 ^abc^	0.79 ± 0.13 ^cd^	13.2 ± 0.9 ^bc^	4.1 ± 0.4 ^cd^	6.1 ± 0.25 ^de^	0.10 ± 0.005 ^bcd^
KF75	21.4 ± 3.31 ^abc^	0.61 ± 0.12 ^de^	14.8 ± 1.2 ^ab^	6.1 ± 0.9 ^b^	7.3 ± 0.35 ^bc^	0.09 ± 0.007 ^cde^
KF50	27.9 ± 2.37 ^a^	1.43 ± 0.12 ^b^	12.9 ± 0.7 ^bc^	4.8 ± 0.4 ^bc^	7.7 ± 0.17 ^b^	0.07 ± 0.003 ^ef^
KF0	20.0 ± 2.41 ^c^	0.93 ± 0.01 ^c^	15.5 ± 0.4 ^a^	4.6 ± 0.2 ^bc^	8.7 ± 0.19 ^a^	0.13 ± 0.004 ^a^
KB100	20. 5 ± 1.13 ^bc^	0.51 ± 0.13 ^e^	11.9 ± 1.4 ^c^	8.0 ± 0.7 ^a^	6.7 ± 0.61 ^cd^	0.08 ± 0.007 ^de^
KB75	20.8 ± 0.82 ^bc^	0.48 ± 0.05 ^e^	11.2 ± 0.5 ^c^	4.5 ± 0.3 ^bc^	6.6 ± 0.14 ^de^	0.07 ± 0.003 ^f^
KB50	22.3 ± 0.38 ^abc^	2.13 ± 0.03 ^a^	12.7 ± 0.5 ^bc^	4.0 ± 0.7 ^cd^	5.7 ± 0.09 ^e^	0.10 ± 0.003 ^bc^
KB0	27.1 ± 1.27 ^ab^	1.23 ± 0.03 ^b^	16.0 ± 0.1 ^a^	2.8 ± 0.7 ^d^	8.7 ± 0.07 ^a^	0.11 ± 0.001 ^b^

^1^ KF#: treatments with chemical fertilization only; KB#: treatments with chemical fertilization and inoculation with *A. calcoaceticus* UTMR2; #: potassium fertilization dose (%) based on the recommended dose for tomatillo cultivation [20]. Different superscript letters indicate a significant difference between the treatments according to the HSD Tukey test (*p* < 0.05, n = 3).

**Table 2:** Physiological and plant-growth parameters in the evaluation of the biofertilizing effect of *Acinetobacter calcoaceticus* UTMR2 in tomatillo plants (*Physalis ixocarpa* Brot. cv. “cáscara morada”) at different doses of potassium.

Treatment ^1^	Photosynthesis (µmol CO_2_/m^2^ s) ^1^	Chlorophyll (mmol/m^2^)	Dry Weight (g)	Plant Height (cm)	Stem Thickness (mm)
KF100	16.1 ± 4.0 ^a^	134.9 ± 3.4 ^bc^	58.0 ± 1.2 ^d^	111.2 ± 8.2 ^b^	10.7 ± 0.1 ^a^
KF75	12.4 ± 3.3 ^a^	125.1 ± 13.8 ^c^	42.6 ± 1.4 ^f^	106.2 ± 7.7 ^b^	10.2 ± 1.0 ^a^
KF50	13.2 ± 2.6 ^a^	133.1 ± 6.2 ^bc^	53.2 ± 1.4 ^e^	112.8 ± 9. 7 ^b^	12.1 ± 0.8 ^a^
KF0	12.3 ± 2.7 ^a^	129.9 ± 11.6 ^bc^	61.1 ± 0.9 ^c^	97.7 ± 4.9 ^b^	10.9 ± 1.2 ^a^
KB100	14.4 ± 2.4 ^a^	133.3 ± 13.6 ^bc^	58.8 ± 0.6 ^cd^	101.8 ± 6.1 ^b^	11.5 ± 0. 7 ^a^
KB75	11.8 ± 0.4 ^a^	152.8 ± 7.2 ^ab^	56.2 ± 0.2 ^d^	112.2 ± 7.1 ^b^	11.0 ± 0.7 ^a^
KB50	13.1 ± 2.2 ^a^	164.6 ± 9.8 ^a^	79.8 ± 0.5 ^a^	142.2 ± 4.7 ^a^	11.5 ± 1.4 ^a^
KB0	12.8 ± 2.5 ^a^	176.9 ± 4.9 ^a^	65.2 ± 0.9 ^b^	104.8 ±5.3 ^b^	11.3 ± 0.8 ^a^

^1^ KF#: treatments with chemical fertilization only; KB#: treatments with chemical fertilization and inoculation with *A calcoaceticus* UTMR2; #: potassium fertilization dose (%) based on the recommended dose for tomatillo cultivation [20]. Different superscript letters indicate a significant difference between the treatments according to the HSD Tukey test (*p* < 0.05, n = 3).

**Table 3:** Fruit yield parameters in the evaluation of the biofertilizing effect of *Acinetobacter calcoaceticus* UTMR2 in tomatillo plants (*Physalis ixocarpa* Brot. cv. “cáscara morada”) at different doses of potassium.

	Yield				Diameter	
Treatment ^1^	Fruit Yield per Plant (g/plant)	Number of Fruits per Plant	Fruit Weight (g/fruit)	Yield Index	Polar (cm)	Equatorial (cm)
KF100	270.1 ± 5.5 ^b^	14.5 ± 0.3 ^a^	18.6 ± 0.2 ^cd^	2.69± 0.04 ^b^	3.5 ± 0.28 ^a^	4.1 ± 0.34 ^a^
KF75	276.7 ± 4.5 ^ab^	13.0 ± 0.4 ^abc^	21.5 ± 0.8 ^a^	2.75 ± 0.03 ^ab^	3.7 ± 0.22 ^a^	4.5 ± 0.11 ^a^
KF50	279.1 ± 11.5 ^ab^	13.5 ± 1.2 ^ab^	20.6 ± 1.1 ^ab^	2.73 ± 0.07 ^ab^	3.5 ± 0.20 ^a^	4.4 ± 0.17 ^a^
KF0	215.3 ± 5.6 ^c^	12.2 ± 0.5 ^bc^	17.6 ± 0. 5 ^d^	2.31 ± 0.04 ^c^	3.3 ± 0.13 ^a^	4.1 ± 0.14 ^a^
KB100	229.0 ± 7.1 ^c^	11.6 ± 0. 7 ^c^	19.8 ± 0. 6 ^abc^	2.41 ± 0.04 ^c^	3.5 ± 0.27 ^a^	4.3 ± 0.22 ^a^
KB75	296.5 ± 8.6 ^a^	14.2 ± 0.4 ^a^	20.9 ± 0.1^ab^	2.86 ± 0.06 ^a^	3.4 ± 0.15 ^a^	4.3 ± 0.30 ^a^
KB50	230.2 ± 10.9 ^c^	11.9 ± 0.4 ^bc^	19.3 ± 0.3 ^bcd^	2.42 ± 0.07 ^c^	3.4 ± 0.27 ^a^	4.3 ± 0.17 ^a^
KB0	287.2 ± 9.4 ^ab^	14.6 ± 0.4 ^a^	19.8 ± 1.1 ^abc^	2.80 ± 0.06 ^ab^	3.5 ± 0.02 ^a^	4.3 ± 0.39 ^a^

^1^ KF#: treatments with chemical fertilization only; KB#: treatments with chemical fertilization and inoculation with *A. calcoaceticus* UTMR2; #: potassium fertilization dose (%) based on the recommended dose for tomatillo cultivation [20]. Different superscript letters indicate a significant difference between the treatments according to the HSD Tukey test (*p* < 0.05, n = 3).

**Table 4:** Physicochemical parameters of the tomatillo fruits in the evaluation of the biofertilizing effect of *Acinetobacter calcoaceticus* UTMR2 in tomatillo plants (*Physalis ixocarpa* Brot. cv. “cáscara morada”) at different doses of potassium.

Treatment ^1^	Total Soluble Solids (°Brix)	Titratable Acidity (%)	pH	Maturity Index	Ash (%)	Protein (%)	Fiber (%)	Moisture (%)	Fat (%)	Electric Conductivity (dS/m)
KF100	6.0 ± 0.01 ^ab^	0.83 ± 0.01 ^b^	3.90 ± 0.006 ^c^	7.3 ± 0.13 ^d^	0.64 ± 0.03 ^a^	1.1 ± 0.11 ^bc^	1.7 ± 0.07 ^abc^	91.3 ± 0.40 ^c^	0.76 ± 0.03 ^ab^	2.56 ± 0.11 ^a^
KF75	6.1 ± 0.06 ^a^	0.68 ± 0.01 ^c^	3.94 ± 0.006 ^b^	9.0 ± 0.10 ^b^	0.51 ± 0.03 ^b^	2.0 ± 0.21 ^a^	1.2 ± 0.21 ^cd^	91.7 ± 0.32 ^abc^	0.85 ± 0.06 ^a^	2.81 ± 0.72 ^a^
KF50	5.9 ± 0.06 ^b^	0.59 ± 0.01 ^d^	4.07 ± 0.006 ^a^	9.9 ± 0.06 ^a^	0.56 ± 0.03 ^ab^	1.1 ± 0.06 ^bc^	1.0 ± 0.06 ^d^	92.4 ± 0.51 ^a^	0.86 ± 0.01 ^a^	3.29 ± 0.10 ^a^
KF0	5.9 ± 0.01 ^ab^	0.94 ± 0.01 ^a^	3.82 ± 0.006 ^e^	6.3 ± 0.03 ^e^	0.53 ± 0.03 ^ab^	1.4 ± 0.33 ^ab^	1.8 ± 0.09 ^ab^	92.4 ± 0.34 ^ab^	0.58 ± 0.09 ^bc^	2.61 ± 0.13 ^a^
KB100	6.0 ± 0.01 ^ab^	0.60 ± 0.01 ^d^	3.89 ± 0.006 ^c^	10.0 ± 007 ^a^	0.63 ± 0.03 ^a^	1.8 ± 0.25 ^a^	1.9 ± 0.15 ^a^	91.9 ± 0.15 ^abc^	0.76 ± 0.04 ^ab^	2.52 ± 0.01 ^a^
KB75	5.8 ± 0.01 ^bc^	0.93 ± 0.01 ^a^	3.84 ± 0.001 ^d^	6.2 ± 0.05 ^e^	0.53 ± 0.06 ^ab^	1.7 ± 0.21 ^a^	1.3 ± 0.30 ^bcd^	92.4 ± 0.16 ^a^	0.81 ± 0.01 ^a^	3.14 ± 0.59 ^a^
KB50	5.6 ± 0.10 ^cd^	0.61 ± 0.01 ^d^	3.85 ± 0.001 ^d^	9.3 ± 0.27 ^b^	0.63 ± 0.04 ^a^	0.8 ± 0.28 ^c^	2.1 ± 0.33 ^a^	91.8 ± 0.31 ^abc^	0.52 ± 0.13 ^c^	3.48 ± 0.71 ^a^
KB0	5.5 ± 0.23 ^d^	0.70 ± 0.01 ^c^	3.96 ± 0.006 ^b^	7.8 ± 0.27 ^c^	0.63 ± 0.54 ^a^	0.6 ± 0.19 ^c^	2.1 ± 0.03 ^a^	91.5 ± 0.10 ^bc^	0.38 ± 0.10 ^c^	2.64 ± 0.06 ^a^

^1^ Percentages in fresh-weight basis (g/100 g fw). KB#: treatments with chemical fertilization and inoculation with *A. calcoaceticus* UTMR2; #: potassium fertilization dose (%) based on the recommended dose for tomatillo cultivation [20]. Different superscript letters indicate a significant difference between the treatments according to the HSD Tukey test (*p* < 0.05, n = 3).

**Table 5:** Tentative identification of chemical markers in ESI-mode in the evaluation of the biofertilizing effect of *Acinetobacter calcoaceticus* UTMR2 in tomatillo fruits (*Physalis ixocarpa* Brot. cv. “cáscara morada”) at 75% of the recommended potassium dose [20].

RT (min)	m/z (Da)	FC KB75/KF75	*p*. Value	Name	Adduct	Mass Error (ppm)
4.29	683.2535	15.361	0.001397	4,4″-bis(N-feruloyl)serotonin	[M-H_2_O-H]^−^	4
0.5	555.1149	8.0253	0.000612	Salvianolic acid K	[M-H]^−^	1
0.45	241.011	0.46791	0.009537	Glucose 6-phosphate	[M-H_2_O-H]^−^	1
1.37	111.008	0.43534	0.011994	Citraconic acid	[M-H_2_O-H]^−^	2
0.63	173.0085	0.43303	0.033027	Citric acid	[M-H]^−^	1
0.54	170.045	0.43188	0.008629	N-Acetylglutamic acid	[M-H_2_O-H]^−^	2
0.67	85.0286	0.39807	0.017379	2-Hydroxybutyric acid	[M-H_2_O-H]^−^	4
0.48	146.0455	0.3797	0.000548	L-Glutamic acid	[M-H]^−^	3
0.45	132.0298	0.30059	0.000237	L-Aspartic acid	[M-H]^−^	3
3.15	205.0495	0.18011	2.41 × 10^−7^	Sinapic acid	[M-H_2_O-H]^−^	3
0.55	126.0552	0.17595	1.05 × 10^−6^	4-Acetamidobutanoic acid	[M-H_2_O-H]^−^	2
1.83	323.0968	0.17585	1.47 × 10^−7^	Sucrose	[M-H_2_O-H]^−^	3
4.2	609.1436	0.17318	3.20 × 10^−6^	Quercetin 3-*O*-rhamnoside 7-*O*-glucoside	[M-H]^−^	4
3.72	565.2117	0.17204	4.39 × 10^−7^	Magnesium protoporphyrin	[M-H_2_O-H]^−^	4
3.08	193.0498	0.16864	8.50 × 10^−6^	*trans*-Ferulic acid	[M-H]^−^	4
2.27	203.0825	0.15452	6.88 × 10^−6^	L-Tryptophan	[M-H]^−^	1
12.07	293.2106	0.15073	5.64 × 10^−6^	13-L-Hydroperoxylinoleic acid	[M-H_2_O-H]^−^	4
1.38	87.0085	0.14848	2.30 × 10^−5^	Pyruvic acid	[M-H]^−^	3
0.49	473.1517	0.14377	1.33 × 10^−5^	alpha-D-Xylopyranosyl-(1->6)-beta-D-glucopyranosyl-(1->4)-D-glucose	[M-H]^−^	1
0.85	515.1246	0.1309	6.55 × 10^−5^	b-D-Glucuronopyranosyl-(1->3)-a-D-galacturonopyranosyl-(1->2)-L-rhamnose	[M-H]^−^	2
13.88	393.1715	0.11576	0.000477	1,3,8-Trihydroxy-4-methyl-2,7-diprenylxanthone	[M-H]^−^	2

**Table 6:** Identification and quantification of phenolic compounds in the evaluation of the biofertilizing effect of *Acinetobacter calcoaceticus* UTMR2 in tomatillo fruits (*Physalis ixocarpa* Brot cv. “cáscara morada”) at 75% of the recommended potassium dose [20].

Compound	Concentration (µg/g of Dried Sample)	
	Non-Biofertilized (KF75)	Biofertilized (KB75)
Precursor		
Phenylalanine	16.77 ± 0.60 ^a^	16.28 ± 0.09 ^a^
Phenolic acids		
*t*-Cinnamic acid	0.89 ± 0.03 ^a^	0.15 ± 0.01 *^b^
Ferulic acid	2.62 ± 0.16^a^	2.04 ± 0.04 ^b^
Protocatechuic acid	0.17 ± 0.01 *^a^	0.06 ± 0.005 *^b^
Vanillic acid	0.10 ± 0.01 *^a^	0.11 ± 0.003 *^a^
4-Coumaric acid	0.64 ± 0.03 ^a^	0.44 ± 0.01 ^b^
Chlorogenic acid	6.06 ± 0.13 ^a^	9.52 ± 0.12 ^b^
Sinapic acid	1.44 ± 0.04 ^a^	0.93 ± 0.01 ^b^
Flavonoids		
Quercetin-3-glucoside	0.79 ± 0.04 *^a^	0.66 ± 0.01 *^b^
Quercetin 3,4′-di-O-glucoside	107.25 ± 7.34 ^a^	60.68 ± 2.21 ^b^
Rutin	14.85 ± 0.85 ^a^	15.91 ± 0.21 ^a^

* Concentration determined below the limit of quantification. Different superscript letters mean statistical differences among biofertilized and non-biofertilized treatments (*t*-test; *p* < 0.05).

**Table 7:** Experimental treatments to evaluate the biofertilizing effect of *Acinetobacter calcoaceticus* UTMR2 in tomatillo plants (*Physalis ixocarpa* Brot. cv. “cáscara morada”) at different doses of potassium.

Number	Bacterial Inoculant	K Dose in Nutritive Solution	Treatment
T1	No	100%	KF100
T2		75%	KF75
T3		50%	KF50
T4		0%	KF0
T5	Yes	100%	KB100
T6		75%	KB75
T7		50%	KB50
T8		0%	KB0

## Data Availability

All the data generated in this study are available in the article and its Supplementary Materials.

## Supplementary Materials

The following supporting information can be downloaded at https://www.mdpi.com/article/10.3390/plants12030466/s1: Table S1. Color parameters for the tomatillo fruits (*Physalis ixocarpa* Brot. cv. “cáscara morada”) in the evaluation of the biofertilizing effect of *Acinetobacter calcoaceticus* UTMR2 at different doses of potassium. Table S2. Mineral element content in tomatillo fruits (*Physalis ixocarpa* Brot. cv. “cáscara morada”) in the evaluation of the biofertilizing effect of *Acinetobacter calcoaceticus* UTMR2 at different doses of potassium. Table S3. Two-way analysis of variance (ANOVA) for the relevant parameters of tomatillo plants (*Physalis ixocarpa* Brot. cv. “cáscara morada”) in the evaluation of the biofertilizing effect of *Acinetobacter calcoaceticus* UTMR2 at different doses of potassium. Table S4. Two-way analysis of variance (ANOVA) for the relevant parameters of tomatillo fruits (*Physalis ixocarpa* Brot. cv. “cáscara morada”) in the evaluation of the biofertilizing effect of *Acinetobacter calcoaceticus* UTMR2 at different doses of potassium. Table S5. dMRM, mass spectrometric, and quantification conditions for each phenolic compound in the evaluation of the biofertilizing effect of *Acinetobacter calcoaceticus* UTMR2 in tomatillo fruits (*Physalis ixocarpa* Brot. cv. “cáscara morada”) at 75% of the recommended potassium dose. Figure S1. Phenol content and antioxidant capacity in tomatillo fruits (*Physalis ixocarpa* Brot. cv. “cáscara morada”) in the evaluation of the biofertilizing effect of *Acinetobacter calcoaceticus* UTMR2 at different doses of potassium. Figure S2. Scree plot for the percentage of coverage of the PCA analysis in the evaluation of the biofertilizing effect of *Acinetobacter calcoaceticus* UTMR2 in tomatillo plants (*Physalis ixocarpa* Brot. cv. “cáscara morada”) at different doses of potassium.
