# Supplementary material for: Combined Effect of the Potassium Dose and Plant Biofertilization by Acinetobacter calcoaceticus on the Growth, Mineral Content, Nutritional Quality, Antioxidant Activity, and Metabolomic Features of Tomatillo Fruits (Physalis ixocarpa Brot.)
Source: Plants (Basel). 2023 Jan 19;12(3):466. doi: 10.3390/plants12030466 (PMC9920258; doi:10.3390/plants12030466)
Supplement: Supplementary file 1 [file plants-12-00466-s001.zip › plants-2152456-supplementary.pdf]

Supplementary Materials

Combined effect of the potassium dose and plant biofertilization by *Acinetobacter calcoaceticus* on the growth, mineral content, nutritional quality, antioxidant activity and metabolomic features of tomatillo fruits (*Physalis ixocarpa* Brot.).

Plants

Heriberto F. Ramírez-Cariño, Carlos E. Ochoa-Velasco, José A. Guerrero-Analco, Juan L. Monribot-Villanueva, Concepción Calderón-García, Elizabeth González-Terreros, Cirenio Escamirosa-Tinoco, Isidro Morales*, Rogelio Valadez-Blanco*

* Correspondence:

rvaladez@mixteco.utm.mx (R.Valadez-Blanco), Instituto de Agroindustrias, Universidad Tecnológica de la Mixteca, Carretera a Acatlima km. 2.5, Huajuapan de León, Oaxaca 69000, México
imoralesg@ipn.mx (I. Morales), Instituto Politécnico Nacional, Centro Interdisciplinario de Investigación para el Desarrollo Integral Regional - Unidad Oaxaca, Hornos 1003, Santa Cruz Xoxocotlán, Oaxaca 71230, México

SUPPLEMENTARY TABLES

Table S1. Color parameters (CIELAb) for the tomatillo fruits (*Physalis ixocarpa* Brot. cv. "cáscara morada") in the evaluation of the biofertilizing effect of *Acinetobacter calcoaceticus* UTMR2 at different doses of potassium.

**Treatment**[1]	**L***	**a***	**b***
KF100	55.5 ± 1.34 ^{ab}	-5.9 ± 0.91 ^{ab}	24.3 ± 2.16 ^{a}
KF75	55.9 ± 2.88 ^{ab}	-5.6 ± 1.05 ^{a}	24.1 ± 2.62 ^{a}
KF50	56.3 ± 1.59 ^{a}	-6.9 ± 1.21 ^{ab}	27.8 ± 3.08 ^{a}
KF0	53.4 ± 2.73 ^{ab}	-7.2 ± 0.78 ^{ab}	25.8 ± 3.67 ^{a}
KB100	54.2 ± 3.10 ^{ab}	-7.2 ± 0.56 ^{ab}	26.9 ± 2.10 ^{a}
KB75	53.8 ± 2.84 ^{ab}	-6.7 ± 1.09 ^{ab}	24.6 ± 1.26 ^{a}
KB50	51.1 ± 1.44 ^{b}	-7.7 ± 0.78 ^{b}	25.9 ± 2.07 ^{a}
KB0	54.7 ± 3.86 ^{ab}	-6.7 ± 1.63 ^{ab}	24.8 ± 3.60 ^{a}

[1]KF#: Treatments with chemical fertilization only; KB#: Treatments with chemical fertilization and inoculation with *A. calcoaceticus* UTMR2; #: potassium fertilization dose (%) based on the recommended dose for tomatillo cultivation [19]. Different superscript letters indicate a significant difference between the treatments according to the HSD Tukey test ($p < 0.05$, $n = 3$).

Table S2. Mineral-element content (g/kg dw) in tomatillo fruits (*Physalis ixocarpa* Brot. cv. "cáscara morada") in the evaluation of the biofertilizing effect of *Acinetobacter calcoaceticus* UTMR2 at different doses of potassium.

Treatment[1]	K	P	Ca	Na	Mg	Mn
KF100	17.1 ± 5.21 [a]	1.43 ± 0.10 [a]	0.03 ± 0.03 [b]	0.83 ± 0.10 [d]	2.6 ± 0.15 [a]	0.018 ± 0.003 [a]
KF75	18.6 ± 38 [a]	1.40 ± 0.08 [a]	0.09 ± 0.11 [ab]	1.33 ± 0.04 [bc]	2.7 ± 0.09 [a]	0.020 ± 0.003 [a]
KF50	24.2 ± 3.90 [a]	1.34 ± 0.28 [a]	0.16 ± 0.08 [ab]	1.32 ± 0.03 [bc]	2.5 ± 0.25 [a]	0.017 ± 0.002 [a]
KF0	18.8 ± 2.57 [a]	1.38 ± 0.22 [a]	0.28 ± 0.08 [a]	1.52 ± 0.33 [b]	2.8 ± 0.22 [a]	0.017 ± 0.003 [a]
KB100	18.4 ± 1.68 [a]	1.48 ± 0.25 [a]	0.04 ± 0.08 [b]	1.22 ± 0.11 [bcd]	2.6 ± 0.36 [a]	0.020 ± 0.002 [a]
KB75	17.4 ± 1.42 [a]	1.45 ± 0.05 [a]	0.19 ± 0.07 [ab]	0.90 ± 0.17 [cd]	2.7 ± 0.37 [a]	0.011 ± 0.010 [a]
KB50	19.9 ± 1.29 [a]	1.76 ± 0.45 [a]	0.23 ± 0.05 [ab]	2.26 ± 0.18 [a]	3.4 ± 0.54 [a]	0.023 ± 0.005 [a]
KB0	17.0 ± 1.55 [a]	1.65 ± 0.18 [a]	0.04 ± 0.03 [b]	0.87 ± 0.08 [d]	2.9 ± 0.18 [a]	0.016 ± 0.001 [a]

[1]KF#: Treatments with chemical fertilization only; KB#: Treatments with chemical fertilization and inoculation with *A. calcoaceticus* UTMR2; #: potassium fertilization dose (%) based on the recommended dose for tomatillo cultivation [19]. Different superscript letters indicate a significant difference between the treatments according to the HSD Tukey test ($p < 0.05$, $n = 3$).

Table S3. Two-way analysis of variance (ANOVA) for the relevant parameters of tomatillo plants (*Physalis ixocarpa* Brot. cv. "cáscara morada") in the evaluation of the biofertilizing effect of *Acinetobacter calcoaceticus* UTMR2 at different doses of potassium.

SV	DF	Mean squares						
		[K]	[P]	[Ca]	[Na]	[Mg]	Chlorophyll	Dry weight
K dose	3	16.61ns	1.89***	13.33***	6.20***	6.56***	470.84*	329.60***
		(0.07)	(0.0001)	(0.0001)	(0.0001)	(0.0001)	(0.0112)	(0.0001)
Inoculation	1	7.62ns	0.13***	8.02**	0.03ns	2.50***	4108.16***	766.14***
		(0.27)	(0.0008)	(0.0025)	(0.7426)	(0.0001)	(0.0001)	(0.0001)
K dose*Inoculation	3	53.47**	0.29***	4.88**	11.02***	2.21***	615.87***	199.17***
		(0.001)	(0.0001)	(0.0020)	(0.0001)	(0.0001)	(0.0038)	(0.0001)
Error	16							
Total	23							

SV: sources of variation; DF: degrees of freedom; value in parenthesis, level of significance; ns: non-significant value of F ($P > 0.05$), *: significant F value ($P \leq 0.05$), **: highly significant value of F ($P \leq 0.01$), ***: very highly significant value of F ($P \leq 0.001$).

Table S4. Two-way analysis of variance (ANOVA) for the relevant parameters of tomatillo fruits (*Physalis ixocarpa* Brot. cv. "cáscara morada") in the evaluation of the biofertilizing effect of *Acinetobacter calcoaceticus* UTMR2 at different doses of potassium.

SV	DF	Mean squares					
		**Yield index**	**Maturity index**	**Protein**	**Fiber**	**Fat**	**FRAP**
K dose	3	0.088*** (0.0001)	7.46*** (0.0001)	1.15*** (0.0001)	0.52*** (0.0001)	0.13*** (0.0001)	106770.47*** (0.0001)
Inoculation	1	0.00003ns (0.911)	0.26*** (0.0036)	0.19*** (0.0061)	1.11*** (0.0001)	0.13*** (0.0001)	25200.72*** (0.0001)
K dose*Inoculation	2	0.213*** (0.0001)	8.91*** (0.0001)	0.63*** (0.0001)	0.29*** (0.0014)	0.04*** (0.0027)	414287.64*** (0.0001)
Error	16						
Total	23						

SV: sources of variation; DF: degrees of freedom; value in parenthesis: level of significance; ns: non-significant value of F (P > 0.05), *: significant F value (P ≤ 0.05), **: highly significant value of F (P ≤ 0.01), ***: very highly significant value of F (P ≤ 0.001).

Table S5. dMRM, mass spectrometric and quantification conditions for each phenolic compound in the evaluation of the biofertilizing effect of *Acinetobacter calcoaceticus* UTMR2 in tomatillo fruits (*Physalis ixocarpa* Brot. cv. "cáscara morada") at 75% of the potassium recommended dose [19].

Compound	dMRM transition			Mass spectrometric conditions			Quantification conditions		
	Precursor ion	Production	Retention time	Collision energy	Fragmentor	Polarity	Quantification range (µM)	Regression type	R^2
Shikimic acid	173.1	111.1	0.49	10	100	Negative	0.5 - 19	Quadratic	0.99
Gallic acid	169.0	125.2	1.4	10	100	Negative	1 - 19	Quadratic	0.99
L-Phenylalanine	166.1	131.0	1.92	10	100	Positive	0.25 - 19	Quadratic	0.99
Protocatechuic acid	153.0	109.1	2.5	10	100	Negative	0.25 - 19	Quadratic	0.99
4-Hydroxybenzoic acid	137.1	92.8	3.76	10	100	Negative	0.25 - 19	Quadratic	0.99
Gentisic acid	153.0	109.0	3.83	10	100	Negative	0.25 - 19	Quadratic	0.99
4-Hydroxyphenylacetic acid	107.1	77.0	4.72	20	140	Positive	0.25 - 19	Quadratic	0.99
(-)-Epigallocatechin	305.1	125.0	4.83	20	140	Negative	1 - 17	Quadratic	0.99
(+)-Catechin	291.0	138.9	5.07	10	100	Positive	0.5 - 19	Quadratic	0.99
Vanillic acid	169.0	93.0	5.12	10	100	Positive	0.25 - 19	Quadratic	0.99
Scopolin	355.1	193.0	5.25	20	100	Positive	0.25 - 19	Quadratic	0.99
Chlorogenic acid	355.1	163.0	5.34	10	100	Positive	0.25 - 19	Quadratic	0.99
Caffeic acid	181.0	163.	5.38	10	100	Positive	0.5 - 19	Quadratic	0.99
Malvin	655.1	331.1	5.82	40	100	Positive	0.5 - 19	Quadratic	0.99
Kuromanin	449.0	286.9	6.34	30	100	Positive	0.5 - 19	Quadratic	0.99
Procyanidin B2	577.1	425.1	6.4	10	100	Negative	1 - 19	Quadratic	0.99
Vanillin	153.0	124.9	6.52	10	100	Positive	0.25 - 19	Quadratic	0.99
Keracyanin	595.2	287.1	6.88	20	100	Positive	0.5 - 19	Quadratic	0.99
(-)-Epicatechin	291.0	138.8	6.96	10	100	Positive	0.5 - 19	Quadratic	0.99
4-Coumaric acid	165.0	147.0	7.21	10	100	Positive	0.25 - 19	Quadratic	0.99
Mangiferin	423.0	302.8	7.32	10	100	Positive	0.5 - 19	Quadratic	0.99

Umbelliferone	163.0	107.0	7.64	30	100	Positive	0.25 - 19	Quadratic	0.99
(-)-Gallocatechin gallate	458.9	139.0	7.95	20	80	Positive	1 - 19	Quadratic	0.99
Scopoletin	193.0	133.0	8.4	10	100	Positive	0.25 - 19	Quadratic	0.99
Ferulic acid	195.1	145.0	8.6	20	100	Positive	0.25 - 19	Quadratic	0.99
Quercetin 3,4-di-O-glucoside	627.0	302.9	8.77	10	100	Positive	0.5 - 19	Quadratic	0.99
3-Coumaric acid	165.05	147.04	8.81	10	100	Positive	0.5 - 19	Quadratic	0.99
Salicylic acid	137.0	93	9.15	10	100	Negative	0.5 - 19	Quadratic	0.99
Sinapic acid	225.1	207.1	9.16	10	100	Positive	0.25 - 19	Quadratic	0.99
Epicatechin gallate	443.1	123.0	9.83	10	100	Positive	1 - 19	Quadratic	0.99
Ellagic acid	300.5	145.0	9.98	30	170	Negative	1 - 19	Quadratic	0.99
Myricitrin	465.0	318.9	10.03	10	100	Positive	1 – 19	Quadratic	0.99
Pelargonidin	271.1	121	10.22	20	10	Positive	1 - 19	Quadratic	0.97
Quercetin 3-D-galactoside	465.0	302.9	10.26	10	100	Positive	0.25 - 19	Quadratic	0.99
Rutin	611.0	302.9	10.35	10	100	Positive	0.25 - 19	Quadratic	0.99
p-Anisic acid	153.1	109.0	10.45	5	120	Positive	0.25 - 19	Quadratic	0.99
Quercetin 3-glucoside	465.0	303.0	10.57	10	100	Positive	0.25 - 19	Quadratic	0.99
Luteolin 7-O-glucoside	449.0	287.0	10.77	10	100	Positive	0.5 – 19	Quadratic	0.99
Malvidin	331.1	287.1	11.14	20	100	Positive	1 - 17	Quadratic	0.96
2,4-Dimethoxy-6-methylbenzoic acid	197.0	179.0	11.41	5	80	Positive	0.25 - 19	Quadratic	0.99
Penta-O-galloyl-B-D-glucose	771.1	153.0	11.68	20	100	Positive	0.5 - 19	Quadratic	0.99
Kaemperol 3-O-glucoside	449.0	286.9	11.91	10	100	Positive	0.25 - 19	Quadratic	0.99
Quercitrin	449.1	303.1	11.95	10	100	Positive	0.5 - 19	Quadratic	0.99
Naringin	273.0	153.0	12.13	10	120	Positive	0.25 - 19	Quadratic	0.99
Myricetin	317.0	179.0	12.29	10	100	Negative	0.5 - 15	Quadratic	0.99
Hesperidin	609.1	301.1	12.68	20	100	Negative	0.5 - 19	Quadratic	0.99
trans-Resveratrol	229.1	135.0	12.69	10	100	Positive	0.5 - 19	Quadratic	0.99
Rosmarinic acid	361.1	163.0	12.8	10	100	Positive	0.5 - 19	Quadratic	0.99

Secoisolariciresinol	363.2	137.1	13.02	20	100	Positive	0.5 - 19	Quadratic	0.99
Phloridzin	435.0	272.9	13.04	10	100	Negative	0.25 - 19	Quadratic	0.99
trans-Cinnamic acid	149.1	131.0	14.08	10	100	Positive	0.25 - 19	Quadratic	0.99
Psoralen	187.0	131.1	14.99	20	100	Positive	0.25 - 19	Quadratic	0.99
Quercetin	302.9	153.1	15.18	35	100	Positive	1 - 19	Quadratic	0.99
Luteolin	287.1	153.0	15.28	30	100	Positive	0.5 - 19	Quadratic	0.99
Angelicin	187.0	131.1	15.75	20	100	Positive	0.5 - 19	Quadratic	0.99
Naringenin	271.0	151	16.79	10	100	Negative	0.5 - 19	Quadratic	0.99
Apigenin	271.0	153.0	17.45	30	100	Positive	0.5 - 19	Quadratic	0.99
Matairesinol	359.2	137.1	17.55	10	100	Positive	0.25 - 19	Quadratic	0.99
Kaempferol	287.1	153.0	17.81	30	100	Positive	0.25 - 19	Quadratic	0.99
Hesperetin	303.1	177.1	18.06	20	100	Positive	0.25 - 19	Quadratic	0.99
Podophyllotoxin	415.1	397.1	19.01	10	100	Positive	0.25 - 19	Quadratic	0.99
Methyl cinnamate	163.1	131.0	21.46	6	100	Positive	0.25 - 1	Quadratic	0.99
Nordihydroguaiaretic acid	303.0	193.1	22.72	10	100	Positive	0.5 - 19	Quadratic	0.99
Chrysin	255.1	153.0	22.89	40	100	Positive	0.25 - 19	Quadratic	0.99
Kaempferide	301.0	258.2	24.38	20	100	Positive	0.5 - 19	Quadratic	0.99
Emodin	269.0	225.0	27.45	20	150	Negative	1 - 17	Quadratic	0.99
Chrysophanol	255.1	153.0	31.34	40	100	Positive	0.25 - 19	Quadratic	0.99

The retention time variation allowed for the search of the compounds were 2 min in each case. The cell accelerator voltage was 7 V for each compound. Dilutions were made if the concentration of some compounds were higher than the linearity range.

SUPPLEMENTARY FIGURES

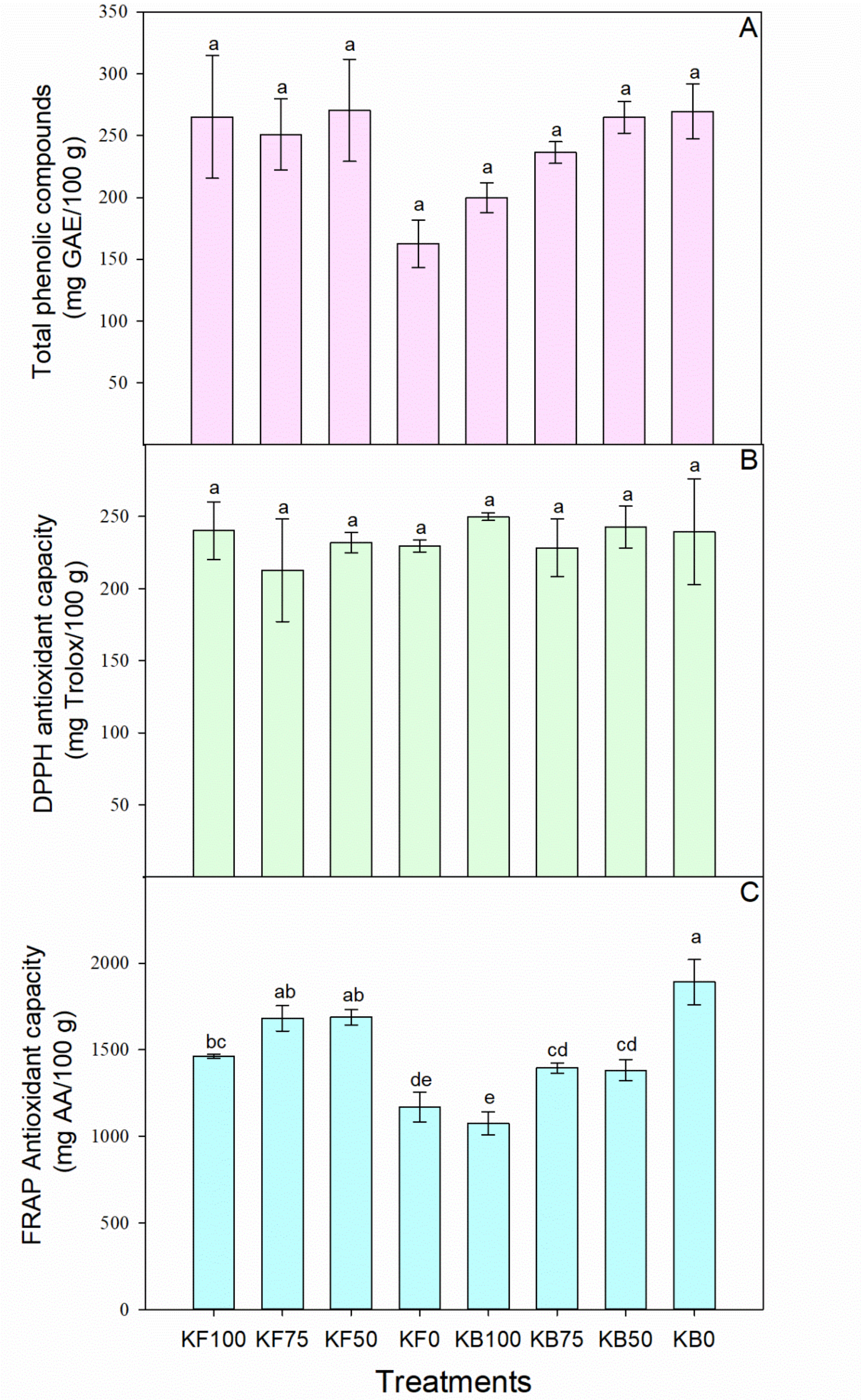

Figure S1. Phenols content and antioxidant capacity in tomatillo fruits (*Physalis ixocarpa* Brot. cv. "cáscara morada") in the evaluation of the biofertilizing effect of *Acinetobacter calcoaceticus* UTMR2 at different doses of potassium. KF#: Treatments with chemical fertilization only; KB#: Treatments with chemical fertilization and inoculation with *Acinetobacter calcoaceticus* UTMR2; #: potassium fertilization dose (%) based on the recommended dose for tomatillo cultivation [19]. Different superscript letters indicate a significant difference between the treatments. GAE: gallic acid equivalent; AA: ascorbic acid.

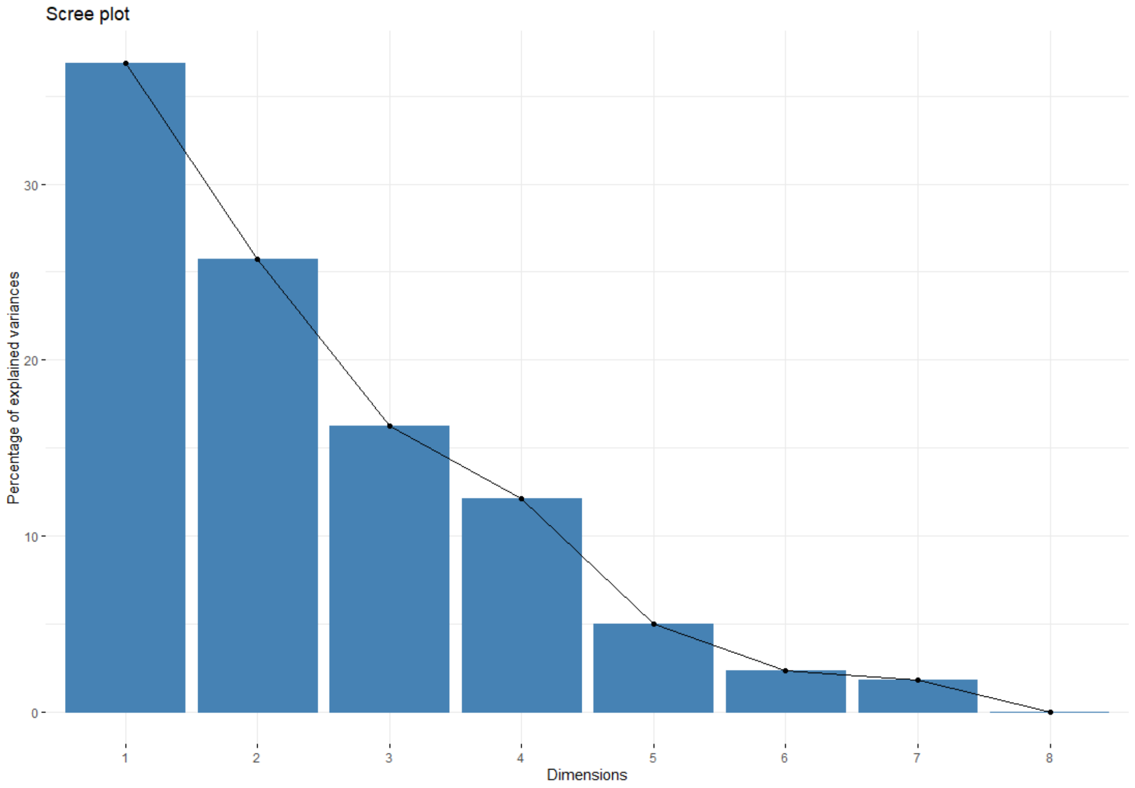

Figure S2. Scree plot for the percentage of coverage of the PCA analysis in the evaluation of the biofertilizing effect of *Acinetobacter calcoaceticus* UTMR2 in tomatillo plants (*Physalis ixocarpa* Brot. cv. "cáscara morada") at different doses of potassium.
